# Hierarchical amino acid utilization and its influence on fermentation dynamics: rifamycin B fermentation using *Amycolatopsis mediterranei *S699, a case study

**DOI:** 10.1186/1475-2859-5-32

**Published:** 2006-11-02

**Authors:** Prashant M Bapat, Debasish Das, Sujata V Sohoni, Pramod P Wangikar

**Affiliations:** 1 Department of Chemical Engineering, Indian Institute of Technology, Bombay, Powai, Mumbai 400 076, India.; 2 Center for Mikrobiel Bioteknologi, BioCentrum-DTU, Danmarks Tekniske Universitet, Bygning 223, DK-2800 Kgs. Lyngby, Denmark.

## Abstract

**Background:**

Industrial fermentation typically uses complex nitrogen substrates which consist of mixture of amino acids. The uptake of amino acids is known to be mediated by several amino acid transporters with certain preferences. However, models to predict this preferential uptake are not available. We present the stoichiometry for the utilization of amino acids as a sole carbon and nitrogen substrate or along with glucose as an additional carbon source. In the former case, the excess nitrogen provided by the amino acids is excreted by the organism in the form of ammonia. We have developed a cybernetic model to predict the sequence and kinetics of uptake of amino acids. The model is based on the assumption that the growth on a specific substrate is dependent on key enzyme(s) responsible for the uptake and assimilation of the substrates. These enzymes may be regulated by mechanisms of nitrogen catabolite repression. The model hypothesizes that the organism is an optimal strategist and invests resources for the uptake of a substrate that are proportional to the returns.

**Results:**

Stoichiometric coefficients and kinetic parameters of the model were estimated experimentally for *Amycolatopsis mediterranei *S699, a rifamycin B overproducer. The model was then used to predict the uptake kinetics in a medium containing cas amino acids. In contrast to the other amino acids, the uptake of proline was not affected by the carbon or nitrogen catabolite repression in this strain. The model accurately predicted simultaneous uptake of amino acids at low cas concentrations and sequential uptake at high cas concentrations. The simulated profile of the key enzymes implies the presence of specific transporters for small groups of amino acids.

**Conclusion:**

The work demonstrates utility of the cybernetic model in predicting the sequence and kinetics of amino acid uptake in a case study involving *Amycolatopsis mediterranei*, an industrially important organism. This work also throws some light on amino acid transporters and their regulation in *A. mediterranei *.Further, cybernetic model based experimental strategy unravels formation and utilization of ammonia as well as its inhibitory role during amino acid uptake. Our results have implications for model based optimization and monitoring of other industrial fermentation processes involving complex nitrogen substrate.

## Background

Majority of the industrial fermentations employ a batch or a fed batch process with complex media that offers multiple substitutable substrates [1]. The batch process goes through several distinct phases of fermentation during the batch cycle. Even small changes in the substrate concentration during the crucial phase of the batch may significantly affect the product yield and quality[2,3]. One of the major nutrient source in a complex medium is the pool of amino acids, peptides and proteins derived from the organic nitrogen substrates such as soybean flour, yeast extract, corn steep liquor, etc. Thus, it is of interest to understand the pattern of uptake of the amino acids during industrially important fermentation processes. The regulation of uptake of nitrogen substrates has been studied extensively in prokaryotes [4-11] and lower eukaryotes such as *Saccharomyces cerevisiae *[12-16] and the filamentous fungus *Aspergillus nidulans *[17-20]. These organisms regulate the amino acid uptake through a multiplicity of amino acid transporters (permeases). Different amino acid transporters differ in their substrate specificities, uptake capacities and the mode of regulation [21]. All fungal and several of the bacterial amino acid transporters show significant sequence similarities, suggesting a unique transporter family conserved across the prokaryotic-eukaryotic boundary [17]. Most of the transporters are specific for one or a few related L-amino acids. In addition, several organisms such as *Saccharomyces cerevisiae, Aspergillus nidulans, Penicillum chrysogenum *and *Neurospora crassa *possess a broad specificity, large capacity, general amino acid permease (GAP) mediating the uptake of most L- and D-amino acids, non proteinogenic amino acids such as citrulline, ornithine and a number of amino acid analogs [21-24]. Most microorganisms thus possess multiple transport systems with partially overlapping specificities.

Although regulation of amino acid transporters in yeast and fungi operates mainly at the level of transcription, post transcriptional, translational and posttranslational regulation has been reported [12,13,16,25,26]. The transcriptional regulation includes nitrogen catabolite repression, carbon catabolite repression and regulation in response to amino acid availability[21]. Many specific permeases in *Saccharomyces cerevisiae *have been reported to be expressed constitutively [27]. However, this is not a general rule for microbial eukaryotes. For example, proline permease encoded by the prn B gene in *Aspergillus nidulans *is highly inducible [21]. Proline can act as both a carbon source and nitrogen source. Thus, the efficiency of prn B expression is highly dependent on the presence of other carbon and nitrogen substrates in the medium, possibly regulated via nitrogen catabolite and carbon catabolite repression. Likewise, the L-serine permease in *Saccharomyces cerevisiae *and *Eschirichia coli *is inducible as L-serine being its only substrate and inducer [10,21]. Its activity is highly regulated by nitrogen sources, with low activity in the presence of ammonia and substantially increased activity in nitrogen starved cells.

Most of the industrial fermentations involving actinomycetes employ a mixture of inorganic and organic nitrogen substrates. For the commercially important actinomycete fermentations, the sequence of uptake of amino acids and the underlying mechanism of regulation has not been reported. It is of interest to predict the sequence of uptake of an amino acid and its implication on product formation under various nitrogen substrate combinations. We have chosen to study the amino acid uptake in a rifamycin B over-producer strain of *Amycolatopsis mediterranei *S699. Rifamycin B is an important antitubercular antibiotic [28] while *Amycolatopsis mediterranei *S699 is an Actinomycete, a species that is a source of a majority of marketed antibiotics [29]. We note that this strain is not an amino acid auxotroph and can grow on ammonia as a sole nitrogen substrate [30,31]. The cybernetic model presented here assumes that the uptake of each amino acid is aided by a key enzyme, which is subject to induction by substrate and nitrogen catabolite repression. Physiologically this enzyme could be an amino acid transporter or permease. Through a model-driven experimental analysis we address the following key questions: (i) what is the stoichiometry and sequence of amino acid uptake in a batch fermentation of *Amycolatopsis mediterranei? *(ii) is the sequence of uptake dependent on the amino acid abundance in the medium? (iii) what is the likely multiplicity of the transporters in *Amycolatopsis mediterranei? *(iv) how the presence of different nitrogen substrates affect product formation?

## Results

Amino acids can be assimilated as sole source of carbon and nitrogen during the microbial growth. To verify this with our model strain *Amycolatopsis mediterranei *S699, we set up preliminary growth experiments with amino acids mixture with or without glucose as a carbon source. First, we studied the pattern of uptake of amino acids in batch fermentations with (i) a defined medium containing 3.25 mM of each of the 20 amino acids being the sole source of carbon and nitrogen and (ii) medium with amino acids as in (i) along with 80 g.l^-1 ^glucose. The concentration of amino acids was chosen so as to keep the initial total nitrogen below 1.5 g.l^-1^, as well as to provide glucose and the mixture of amino acids in approximate stoichiometric proportion as carbon and nitrogen substrates respectively. The results of these two experiments were used to obtain the stoichiometric and kinetic parameters. Subsequently, the model was verified on semi synthetic medium containing 80 g.l^-1 ^glucose supplemented with different concentration of cas amino acids.

### Model fit for defined medium

**Figure 1:**
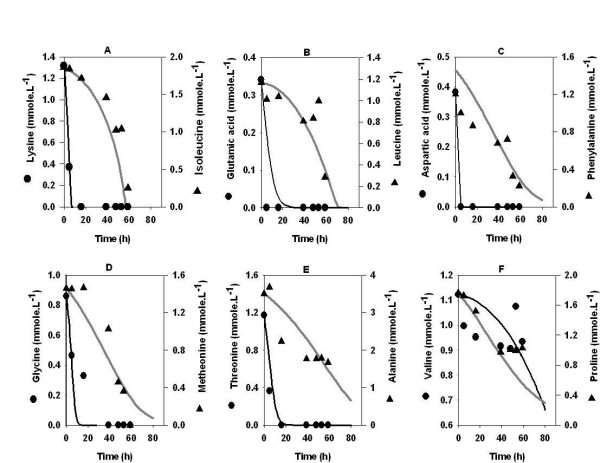
Amino acids uptake profile in medium containing equimolar mixture of amino acids. *Amycolatopsis mediterranei S699 *was cultivated in a media containing mixture of amino acids at a concentration of 3.25 mM each. Besides this, fermentation medium contained, 11 gl^-1 ^CaCO_3_, 1 gl^-1 ^KH_2_PO_4_, 1 gl^-1 ^MgSO_4_, 0.01 gl^-1 ^FeSO_4_, 0.05 gl^-1 ^ZnSO4 and 0.003 gl^-1 ^COCl_2_. For more information, please refer materials and method section.

The results of the experiments with amino acids as the sole source of carbon and nitrogen are presented in figure 1(A–F). We found that although growth occurred in the absence of glucose; rifamycin B was not detected throughout the fermentation batch. In first 20 hrs, lysine, glutamic acid, aspartic acid, glycine and threonine were utilized. This was followed by the utilization of isoluecine, leucine, alanine, valine, phenyl alanine, methionine and proline. The concentration of ammonia in the fermentation broth continued to increase during the course of uptake of amino acids (data not shown). Interestingly, sudden arrest in the utilization of amino acids was observed around 60 hrs. This may possibly be due to the inhibition of growth by the accumulated ammonia (data not shown), a similar observation was reported by Xie and Wang [32] for animal cell cultivation on amino acids.

In industrial fermentation, stoichiometric coefficients are the crucial parameters for the process optimization [33]. From the profiles of glucose, amino acids, CO_2_, biomass and ammonia obtained during shake flask and reactor experiments, we estimated the stoichiometric coefficients of equation 1 and 2. The yield coefficients for amino acids, ammonia and glucose are given in Table 1. The biomass yield coefficient was low when organism utilizes amino acids as a sole source of carbon and nitrogen, whereas it was high where glucose was primary carbon source. The biomass yields on amino acids and ammonia were similar. Interestingly, biomass yield on glucose was significantly influenced by the nitrogen source used. The estimated stoichiometric coefficients are in agreement with the previous reports on *E.coli *and *S.cerevisiae *[34-36].

**Table 1:** Stoichiometric coefficients for batch fermentation of rifamycin B using *Amycolatopsis maditerranei *S699

Stoichiometric coefficients^a^ (moles of substrate. C-mole of biomass^-1^)		
Amino acids	Glucose and amino acids	Glucose and ammonia

Y_1,1,*k *_= 0.51	Y_2,1,*k *_= 0.13	Y_3,2 _= 0.35
Y_1,3 _= 1.30	Y_2,2 _= 0.24	Y_3,6 _= 0.16
Y_1,6 _= 0.85	Y_2,3 _= 1.10	

^a ^*Amycolatopsis mediterranei S699 *was cultivated in two media compositions. (i) Containing mixture of amino acids at a concentraton of 3.25 mM each and (ii) medium (i) supplemented wih glucose (80 g.l^-1^). Both the media were supplemented with 11 gl^-1 ^CaCO_3_, 1 gl^-1 ^KH_2_PO_4_, 1 gl^-1 ^MgSO_4_, 0.01 gl^-1 ^FeSO_4_, 0.05 gl^-1 ^ZnSO4 and 0.003 gl^-1 ^COCl_2_. Samples were taken at regular intervals to estimate concentrations of amino acids, rifamycin B, biomass, residual ammonia and glucose. Online data such as vent CO_2 _and dissolved oxygen was obtained from exit gas analyzer. The offline and online data was further used to estimate stoichiometric coefficients on respective substrate assimilation modes. Y i,j are stoichiometric coefficients where i is reaction number (refer equations in materials and methods) and j is the substrate for example : Amino acid (1) where k represents 20 different amino acids, glucose (2), CO_2 _(3), O_2 _(4), H_2_O (5), Ammonia (6).

The kinetic parameters of the model were estimated by fitting the model equations to amino acid, glucose, biomass as well as product formation profiles. The R^2 ^values, a measure of the goodness of fit of the model, were in the range of 0.90 to 0.95 for amino acid profiles. The specific growth rates measured for the individual amino acids (*μ*_1_^max^) were in the range of 1 × 10^-4 ^to 0.39 h^-1 ^(Table 2). The amino acids which support a higher specific growth rate were utilized first. For example, the *μ*^max ^values for lysine, glutamic acid, aspartic acid, glycine and threonine were much greater than those for isoleucine, leucine, phenylalanine and methionine. The *μ*^max ^for valine was among the lowest indicating strong substrate inhibition; a similar observation was reported by Schimidt and coworkers for a nitrifying bacterium *Nitrosomonas europaea *[37].

**Table 2:** Model parameters for rifamycin B " fermentation using the strain *Amycolatopsis mediterranei*^a^

Model Parameteres^b^															
Amino acids	*μ*_1_^max^	Ks_1_	*k*_*NT*1_	K_I1_	*μ*_2_^max^	Ks_2_	K_I2_	Ks_2,2_	K_I2,2_	*k*_*NT*2_	Kp_2_	K_pI 2_	K_p2 2_	K_pI2,2_	KE1i MathType@MTEF@5@5@+=feaafiart1ev1aaatCvAUfKttLearuWrP9MDH5MBPbIqV92AaeXatLxBI9gBaebbnrfifHhDYfgasaacH8akY=wiFfYdH8Gipec8Eeeu0xXdbba9frFj0=OqFfea0dXdd9vqai=hGuQ8kuc9pgc9s8qqaq=dirpe0xb9q8qiLsFr0=vr0=vr0dc8meaabaqaciaacaGaaeqabaqabeGadaaakeaacqWGlbWsdaWgaaWcbaGaemyrauKaeGymaeZaaSbaaWqaaiabdMgaPbqabaaaleqaaaaa@318D@

Alanine	0.0015	0.0016	0.0259	0.0158	0.0386	2 × 10^-6^	0.51	0.000155	0.0929	0.5929	1.12 × 10^-5^	0.0112	0.1124	1.1236	7.782 × 10^-05^
Glycine	0.0241	0.0004	0.8507	0.1551	0.02	0.003866	0.1543	0.003866	0.1507	0.1507	1.33 × 10^-5^	0.0133	0.1333	1.3333	0.001899
Valine	0.0001	5.15 × 10^-6^	0.5671	0.4738	0.02	5.15 × 10^-6^	0.4382	5.15 × 10^-6^	0.5671	0.5671	8.55 × 10^-6^	0.0085	0.0855	0.8547	8.963 × 10^-06^
Leucine	0.0004	9.5 × 10^-6^	0.0387	0.1583	0.024	1.56 × 10^-5^	0.7835	1.56 × 10^-5^	0.4888	0.0387	7.63 × 10^-6^	0.0076	0.0763	0.7634	4.137 × 10^-05^
Isoleucine	0.0057	2.88 × 0^-5^	0.0323	0.0008	0.0487	2.88 × 10^-5^	0.3545	0.00082	0.1783	0.1783	8.4 × 10^-6^	0.0076	0.0763	0.7634	0.0004403
Threonine	0.0486	0.0011	0.1649	0.0018	0.0259	0.002746	0.5618	0.00171	0.1649	0.1649	9.52 × 10^-6^	0.0084	0.084	0.8403	0.0031509
Serine	0.0191	0.0187	0.6791	0.1634	0.045	0.018685	0.936	0.00018	0.01	0.6791	8.7 × 10^-6^	0.0095	0.0952	0.9524	0.0014994
Proline	0.0101	0.0071	0.1103	0.092	0.06	3.0 × 10^-4^	0.45	0.00057	0.9103	0.9103	7.52 × 10^-6^	0.0087	0.087	0.8696	4.724 × 10^-06^
Aspertic acid	0.0106	5.17 × 10^-7^	0.0272	0.9714	0.052	0.05171	0.0271	5.17 × 10^-9^	0.1609	0.1609	6.71 × 10^-6^	0.0075	0.0752	0.7519	0.0085576
Methionine	0.0091	0.0007	0.0476	0.0404	0.1	7.42 × 10^-5^	0.9429	0.00142	0.0007	0.4758	6.8 × 10^-6^	0.0067	0.0671	0.6711	6.855 × 10^-05^
Glutiamic acid	0.0075	0.0006	0.4581	0.1383	0.075	0.013622	0.0283	1.36 × 10^-5^	0.3058	0.3058	6.06 × 10^-6^	0.0068	0.068	0.6803	0.0018939
Phenylalanine	0.0042	2.26 × 10^-5^	0.0082	0.045	0.0602	2.26 × 10^-6^	0.925	2.26 × 10^-6^	0.0419	0.0082	6.84 × 10^-6^	0.0061	0.0606	0.6061	6.98 × 10^-05^
Glutamine	0.0001	3.59 × 10^-5^	0.033	0.0068	3.51 × 10^-5^	3.59 × 10^-5^	0.0007	3.59 × 10^-5^	0.033	0.033	6.85 × 10^-6^	0.0068	0.0684	0.6845	5.523 × 10^-07^
Lysine	0.0109	1.62 × 10^-7^	0.0636	0.0581	0.0798	0.016222	0.013	0.00015	0.8362	0.8362	6.45 × 10^-6^	0.0068	0.0685	0.6849	0.0040591
Histidine	0.0157	8.79 × 10^-6^	0.0472	0.0942	0.0685	8.79 × 10^-9^	0.4009	8.79 × 10^-6^	0.7243	0.7243	5.52 × 10^-6^	0.0065	0.0645	0.6452	0.0036322
Tyrosine	0.0116	0.0001	0.0567	0.0151	0.0722	0.000151	0.5095	0.00015	0.0367	0.0367	4.9 × 10^-6^	0.0065	0.0552	0.5525	0.0016529
Tryptophan	0.3919	1.49 × 10^-5^	0.0241	0.0049	0.0392	1.49 × 10^-7^	0.4902	1.49 × 10^-5^	0.0241	0.0241	8.7 × 10^-16^	0.0049	0.049	0.4902	0.0100978

^a ^The model parameters were obtained by fitting experimental data (batches shown in Figure 1 and 2) to the model using fmincon subroutine from Matlab.

^b ^Unit of and are in h^-1^. Unit of rest of the parameters are in moles.L^-1^. The value of qp2max⁡
 MathType@MTEF@5@5@+=feaafiart1ev1aaatCvAUfKttLearuWrP9MDH5MBPbIqV92AaeXatLxBI9gBaebbnrfifHhDYfgasaacH8akY=wiFfYdH8Gipec8Eeeu0xXdbba9frFj0=OqFfea0dXdd9vqai=hGuQ8kuc9pgc9s8qqaq=dirpe0xb9q8qiLsFr0=vr0=vr0dc8meaabaqaciaacaGaaeqabaqabeGadaaakeaacqWGXbqCdaqhaaWcbaGaemiCaaNaeGOmaidabaGagiyBa0MaeiyyaeMaeiiEaGhaaaaa@34C5@ has been found to be 0.004 h^-1 ^for all amino acids.

**Figure 2:**
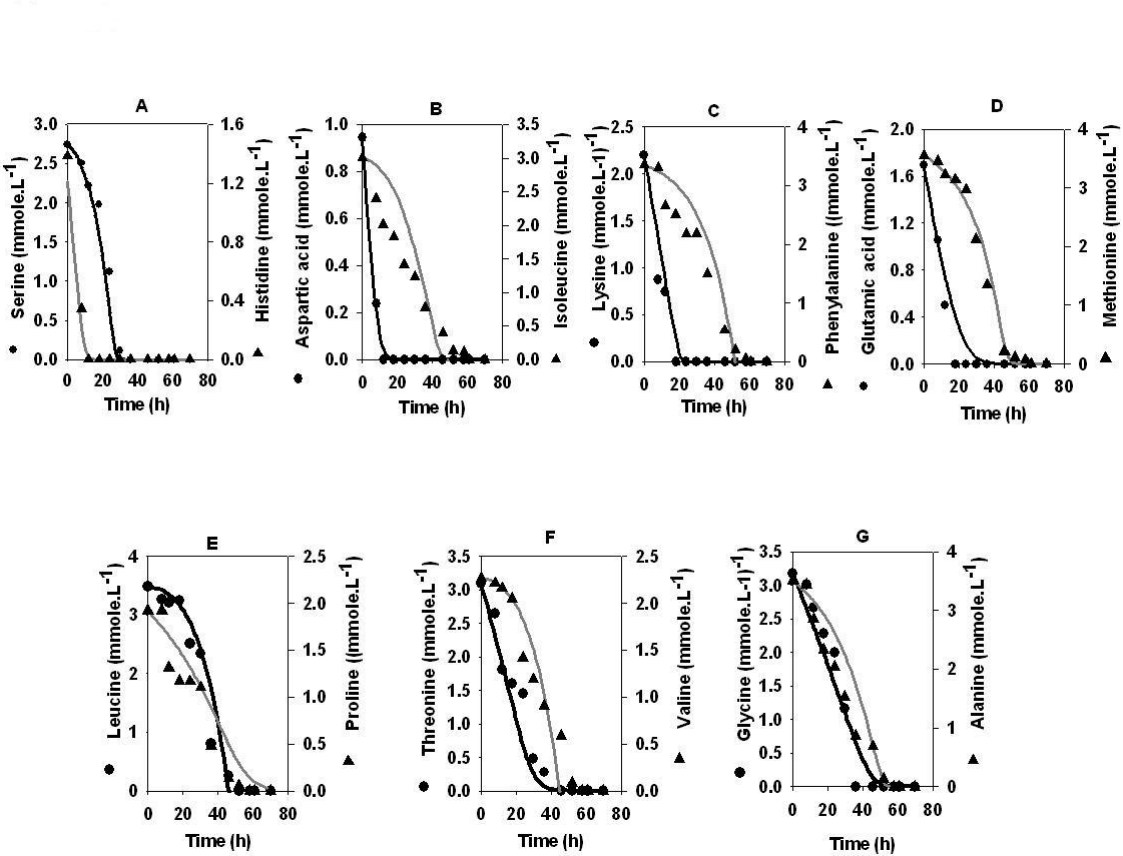
Amino acids uptake profile in medium containing amino acids and glucose. The medium described in legend to figure 1 was supplemented with glucose (80 g.l^-1^). Lines indicate model predictions.

The amino acids uptake profile was then studied in the presence of glucose as a primary carbon substrate (Figure 2). The organism can utilize (i) amino acid as the sole substrate of carbon thereby leading to ammonia accumulation (equ 1), (ii) amino acid along with glucose (equ 2) and (iii) glucose along with ammonia that may be formed as a product of reaction 1 (equ 3). We found that the organism takes up amino acids as the sole substrate for the first 20 hours. Model predictions for the amino acids uptake profile shows a reasonably good fit with the experimental data. Specifically, histidine, aspartic acid, lysine, glutamic acid and threonine were taken up first as observed in the case where amino acids were the sole source of carbon and nitrogen. Ammonia accumulation was detected during this period at a maximum concentration of 0.010 moles of ammonia.l^-1^(data not shown). Subsequently, growth reactions proceed via reaction 2 and 3 with simultaneous uptake of amino acids, ammonia and glucose. During this period, ammonia, serine, isoleucine, phenylalanine, methionine, leucine, proline, valine, glycine and alanine were taken up simultaneously and exhausted by 60 hours leading to nitrogen limitation in the batch. Rifamycin B production was concomitant with the utilization of glucose (data not shown). Finally, 0.035 moles.l^-1 ^rifamycin B was formed, while 0.28 moles.l^-1 ^glucose remain unutilized in this batch.

### Model validation on semi synthetic media

**Figure 3:**
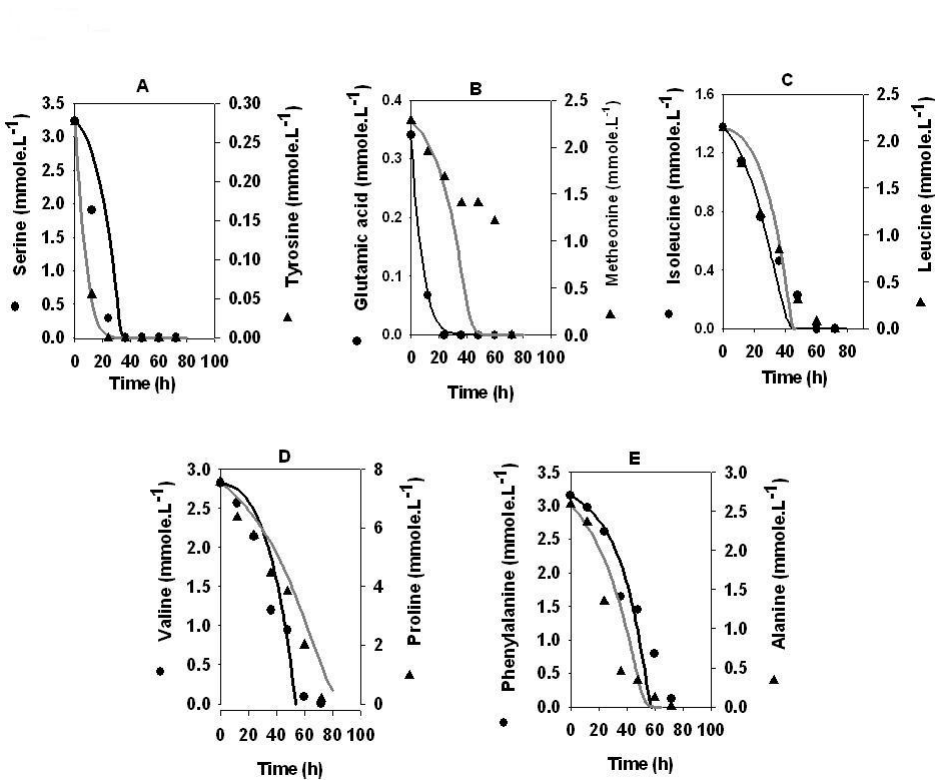
Validation of model on semi defined medium. *Amycolatopsis mediterranei *S699 was cultivated in a medium containing cas amino acid (5 g.l^-1^) and glucose (80 g.l^-1^). The lines represent model predictions. Other media components were same as reported in legends of figure 1.

**Figure 4:**
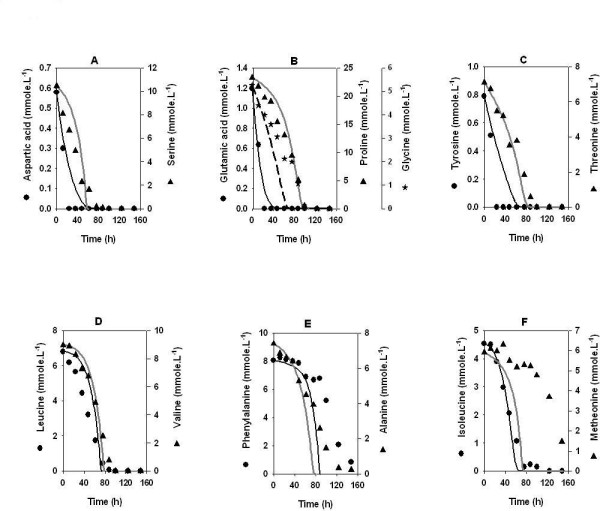
Effect of high concentration of nitrogen on amino acid uptake. *Amycolatopsis mediterranei *S699 was cultivated in a medium containing cas amino acid (15 g.l^-1^) and glucose (80 g.l^-1^). The lines represent model predictions. Other media components were same as reported in legends of figure 1.

The model predictions were experimentally validated on glucose minimal medium supplemented with 5 g.l^-1 ^cas amino acids (Figure 3) and 15 g.l^-1 ^cas amino acids (Figure 4). Note that the relative proportions of the different amino acids in cas are significantly different from that in the synthetic medium described above. The model was able to accurately predict the uptake pattern of amino acids in both the cases. Similar to the synthetic medium, the first 20 hours of the batch were marked by the uptake of amino acids as the sole substrate followed by the simultaneous uptake of amino acids, ammonia and glucose. For 5 g.l^-1 ^cas amino acids, model accurately predicted the uptake profile for almost all amino acids except methionine. As observed from the Figure 3B, model tends to over-estimate the utilization of methionine after 40 hrs. On the other hand, for 15 g.l^-1 ^cas amino acids, the model predicted values deviated from the experimentally observed uptake of phenylalanine, alanine (after 60 hrs), and methionine (after 20 hrs) (Figures 4E and 4F). It may be noted that the cas amino acids contain a relatively higher amount of proline than that used in the defined medium. For 5 g.l^-1 ^cas amino acids, although the amino acids got exhausted in the same sequence as in the defined medium, the utilization of all the amino acids started simultaneously within the first 10 hrs of the batch cycle. Interestingly for 15 g.l^-1 ^cas amino acids (Figure 4) the uptake of amino acids followed a pattern similar to those in the synthetic medium and 5 g.l^-1 ^cas amino acids, albeit with longer lag periods for the utilization of some of the amino acids. Specifically, the utilization of neutral amino acids, glycine, alanine, valine, leucine and isoleucine started after a lag of 20–50 hrs while phenylalanine and methionine (Figures 4E and 4F) showed a lag of 80–100 hrs. Interestingly, proline (Figure 4B) was taken up simultaneously along with the other amino acids and glucose without any lag phase and was completely utilized by 100 hrs.

### Potential multiplicity of amino acid transporters and their regulation

**Figure 5:**
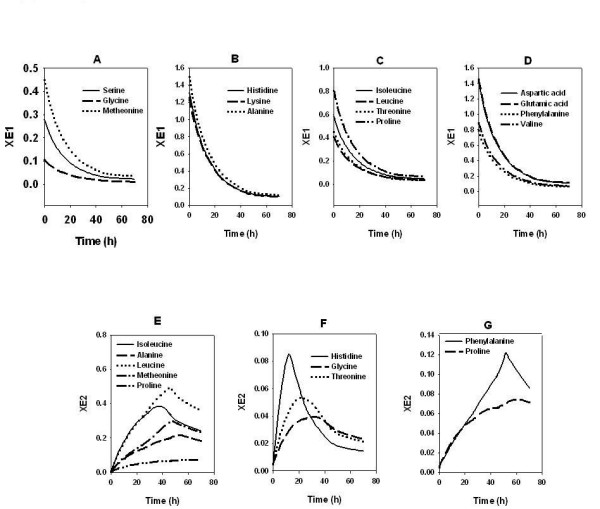
Multiplicity of amino acid transporters. The model was used to simulate induction profiles of the key enzymes responsible for the uptake of amino acids in the medium containing equimolar mixture of amino acids (3.25 mM each) and glucose (80 g.l^-1^). For details refer results and discussion section.

A key latent parameter in the cybernetic model is the relative concentration of the enzyme X_Ei _responsible for the uptake of the amino acid 'i'. The model assumes an independent enzyme to be responsible for the uptake of each amino acid. Physiologically this enzyme could potentially be the respective transporter protein. As observed from the simulated profiles of the transporter proteins, histidine was induced in the early stage of the fermentation (Figure 5F). This was followed by the induction of glycine and threonine transporters. Neutral amino acid transporters of alanine, leucine, isoleucine, methionine and valine (Figure 5E) were the third in the sequence, while the transporter of phenylalanine was induced at the end (Figure 5G). Proline transporter appears to be expressed throughout the batch cycle. A similar trend was observed for 5 g.l^-1 ^cas (data not shown) and 15 g.l^-1 ^cas (data not shown) with the induction in 15 g.l^-1 ^cas being delayed by 20–30 hrs as compared to that in 5 g.l^-1 ^cas.

The enzyme (X_Ei_) profiles for glycine and threonine (Figure 5F) were similar, suggesting a common transporter for the two amino acids. Likewise, the X_Ei _profiles of alanine, leucine, isoleucine, methionine and valine (Figure 5E) were similar suggesting the presence of a common neutral amino acid transporter. Further, we may conjecture that separate transporters exist for proline and phenylalanine. The transporter for phenylalanine was severely repressed by the nitrogen catabolite repression and thus phenylalanine was the last amino acids to be taken up.

## Discussion

Industrial fermentation media are often supplemented with the organic nitrogen substrates, which provide a pool of amino acids in varying proportions. Amino acids not only act as building blocks for the biomass but also play a significant role in the biosynthesis of commercially important metabolites such as antibiotics and therapeutic proteins. The availability of different amino acids and varied cellular preferences for them can affect the antibiotic production to great extent as demonstrated earlier [38,39]. Previously, we have reported a cybernetic model to account for the effect of amino acids on the growth and product formation in rifamycin B fermentation[40]. However, the pool of amino acids was considered as a single substrate. Here, we extend the model by considering each amino acid as an independent substrate. The model accounts for the various mechanisms of regulation of amino acid uptake such as substrate inhibition (Ki) and inhibition from other amino acids (K_NT_) in addition to glucose inhibition. To the best of our knowledge this is the first report where a mathematical model has been developed to predict the sequence of the uptake of amino acids. The model is based on the cybernetic principles and formulated by using the stoichiometry and kinetics of the fermentation.

We show that the amino acids play the role of sole substrate for carbon and nitrogen. This is demonstrated with the cultivations in the media containing a defined mixture of amino acids as the sole source of carbon and nitrogen. Further, *Amycolatopsis mediterranei *was found to assimilate amino acids along with glucose, where glucose plays the role of primary carbon substrate. Interestingly, we found that even in the presence of glucose, the micro-organism utilized amino acids as the sole substrate for the first 20 hours or so. This was marked by the accumulation of ammonia in the extracellular medium. Subsequently, the organism switches to simultaneous uptake of glucose, ammonia and amino acids. This was in agreement with our previous report [33]. The sequence of uptake of amino acids was largely unchanged regardless of the presence of glucose as the carbon substrate. We found that glucose had a substantial effect on the growth, both in terms of the stoichiometric coefficients and specific growth rates. For example, the specific growth rates were ten times lesser whereas biomass yield coefficient was almost five times lesser in the absence of glucose as compared to that with glucose (Table 2).

The model parameters were validated by using the semi defined media. We chose two different concentration levels of cas amino acids for this purpose. The rationale behind this was to check whether the total nitrogen load as well as the individual amino acid concentrations has any impact on the utilization of amino acid and glucose, and rifamycin B productivity. For example in 5 g.l^-1 ^cas amino acid based media, the hierarchy of amino acid utilization was almost similar to the one observed in the medium containing equimolar amino acids with glucose. In 15 g.l^-1 ^cas amino acid based media, sequential uptake of amino acids was observed which implies the preferential utilization of amino acids. The model was also able to predict the rate of formation of rifamycin B, biomass and ammonia as well as the consumption of glucose and ammonia.

Apart from the predictions on the uptake of substrates, the model has pointed towards the potential multiplicity of amino acid transporters for *Amycolatopsis mediterranei *S699 strain. There are two major categories of the transport systems reported for amino acids: a transport system specific for structurally related amino acid family and a general transport system, shared by a large number of amino acids [21]. Apart from the transport systems mentioned above two more systems; general amino acid permease (GAP) and proline transporter (Prn) are reported to be involved in the transportation of amino acids during nitrogen limiting conditions in yeast as well as in fungi. Based on the model simulation and observed data we deduce that the similar system might exist in *Amycolatopsis mediterranei *S699. Similarly we speculate that the proline transporter and GAP system of *Amycolatopsis mediterranei *S699 are partially alleviated from nitrogen regulation. The speculation might hold true as the strain used in these experiments is the outcome of the mutation program conducted by the Lepetit research laboratory, Italy[41,42].

The here-reported model has many potential applications. For example, the model can be used to formulate the optimal media component to boost the growth in the initial phase of fermentation and product formation in the later phase with feeding of the specific amino acids. Likewise, the model can also be used in deciding quality control norms for the organic nitrogen substrates which play significant role towards antibiotic productivity[40]. We further speculate that the model can be used to classify organisms based on the signature of the amino acid uptake sequence. This may enable a quick phenotypic characterization of the various wild-type and mutant strains.

There are certain limitations in the current model structure. It is assumed that each amino acid was assimilated by a specific amino acid transporter system. However, in reality amino acid transporters may be specific toward more than one amino acid. Additional uptake experiments will be required to estimate the level of the transporter proteins at various time points to decipher the specificity of the trasporters. Further, it is important to mention that the kinetic parameters estimated in the preset work correspond to one of the possible solutions that exist in a vast solution space. This is due to the underdetermined nature of the system. The model parameters can be further fine tuned by additional experiments e.g. uptake rate of radio labeled amino acids or glucose.

## Conclusion

In conclusion, we have shown that the hierarchy exists in the amino acid utilization by *Amycolatopsis mediterranei *and it has significant influence on the rifamycin B productivity. In future, it will be interesting to see how the model can be applied for a complex media such as yeast extract and corn steep liquor where amino acid concentration changes continuously throughout the batch due to the hydrolysis of proteins via extracelluler proteases. Although the here-reported study has been applied to a specific strain of micro-organism, the model has a general structure and can be applied to other organisms given the relevant experimental data for estimation of the model parameters.

## Methods

### Organism, culture cultivation and batch fermentation in reactor

The strain *Amycolatopsis mediterranei *S699 was a kind donation from Prof. Heinz Floss (Washington university, USA) and was grown as described by Kim and coworkers [43]. The preparation of fermentation media as well as fermentation conditions were as per the protocol described earlier [44]. The fermentation media was supplemented with one or more of the following: glucose, mixture of 20 amino acids (3.25 mM each; Hi-Media laboratories, Nashik, India) and cas amino acids (Difco, USA) at concentrations to be specified in subsequent section. Batch cultivations were conducted in Biostat^® ^B5 bioreactor (B.Braun Biotech International, Schwarzenberger, Germany).

### Analytical techniques

The estimation of biomass, glucose, free amino acids and rifamycin B was performed at regular intervals during the fermentation experiments as described earlier[40]. The amino acids were estimated by using EZ-faast™ amino acid derivatization kit (Phenomenex Inc, USA) followed by detection through gas chromatography (Mak instruments, Mumbai, India) as described in previous reports [45,46].

### Model Development

A typical fermentation media used in the antibiotic industries consists of multiple carbon and nitrogen substrates in the form of amino acids (available from free organic nitrogen substrates (ONS)), ammonia and glucose. The substrates may be taken up sequentially or simultaneously. Further, extracelluler proteases synthesized by organism helps in sequestering the amino acids from ONS. In this study, we have used synthetic media consisting of free amino acids with or without glucose. Thus, it is desired that the process model be able to predict the growth, product formation and the sequence of uptake of amino acids. This would first require that the underlying stoichiometry of growth on different amino acids be defined. To this end, we assumed that the organism has access to three categories of substrates combinations; (i) an amino acid i (SAAi
 MathType@MTEF@5@5@+=feaafiart1ev1aaatCvAUfKttLearuWrP9MDH5MBPbIqV92AaeXatLxBI9gBaebbnrfifHhDYfgasaacH8akY=wiFfYdH8Gipec8Eeeu0xXdbba9frFj0=OqFfea0dXdd9vqai=hGuQ8kuc9pgc9s8qqaq=dirpe0xb9q8qiLsFr0=vr0=vr0dc8meaabaqaciaacaGaaeqabaqabeGadaaakeaacqWGtbWudaWgaaWcbaGaemyqaeKaemyqae0aaSbaaWqaaiabdMgaPbqabaaaleqaaaaa@31B0@) as the sole source of carbon and nitrogen. The stoichiometry and kinetics of this growth process are represented in equation 1a and 1b respectively. (ii) SAAi
 MathType@MTEF@5@5@+=feaafiart1ev1aaatCvAUfKttLearuWrP9MDH5MBPbIqV92AaeXatLxBI9gBaebbnrfifHhDYfgasaacH8akY=wiFfYdH8Gipec8Eeeu0xXdbba9frFj0=OqFfea0dXdd9vqai=hGuQ8kuc9pgc9s8qqaq=dirpe0xb9q8qiLsFr0=vr0=vr0dc8meaabaqaciaacaGaaeqabaqabeGadaaakeaacqWGtbWudaWgaaWcbaGaemyqaeKaemyqae0aaSbaaWqaaiabdMgaPbqabaaaleqaaaaa@31B0@ as the nitrogen source with *S*_*glc *_as the carbon source (equation 2a and 2b respectively). (iii) Ammonia (*S*_*amm*_) as nitrogen-source and *S*_*glc *_as carbon-source (equation 3a and 3b respectively). Note that with SAAi
 MathType@MTEF@5@5@+=feaafiart1ev1aaatCvAUfKttLearuWrP9MDH5MBPbIqV92AaeXatLxBI9gBaebbnrfifHhDYfgasaacH8akY=wiFfYdH8Gipec8Eeeu0xXdbba9frFj0=OqFfea0dXdd9vqai=hGuQ8kuc9pgc9s8qqaq=dirpe0xb9q8qiLsFr0=vr0=vr0dc8meaabaqaciaacaGaaeqabaqabeGadaaakeaacqWGtbWudaWgaaWcbaGaemyqaeKaemyqae0aaSbaaWqaaiabdMgaPbqabaaaleqaaaaa@31B0@ as the sole source of carbon and nitrogen, the N/C ratio in the substrate is higher than that can be assimilated in the cell mass. As a result, the excess nitrogen is excreted into the media as ammonia-*S*_*amm*_, which can then be utilized by the cells (equation 3a). The product formation requires *S*_*glc *_as the carbon source and is described in equation 4a and 5a with the respective kinetic equations in 4b and 5b. The model assumes that the growth on a given combination of substrates depends on the level of key inducible enzymes XE1i
 MathType@MTEF@5@5@+=feaafiart1ev1aaatCvAUfKttLearuWrP9MDH5MBPbIqV92AaeXatLxBI9gBaebbnrfifHhDYfgasaacH8akY=wiFfYdH8Gipec8Eeeu0xXdbba9frFj0=OqFfea0dXdd9vqai=hGuQ8kuc9pgc9s8qqaq=dirpe0xb9q8qiLsFr0=vr0=vr0dc8meaabaqaciaacaGaaeqabaqabeGadaaakeaacqWGybawdaWgaaWcbaGaemyrauKaeGymaeZaaSbaaWqaaiabdMgaPbqabaaaleqaaaaa@31A7@, XE2i
 MathType@MTEF@5@5@+=feaafiart1ev1aaatCvAUfKttLearuWrP9MDH5MBPbIqV92AaeXatLxBI9gBaebbnrfifHhDYfgasaacH8akY=wiFfYdH8Gipec8Eeeu0xXdbba9frFj0=OqFfea0dXdd9vqai=hGuQ8kuc9pgc9s8qqaq=dirpe0xb9q8qiLsFr0=vr0=vr0dc8meaabaqaciaacaGaaeqabaqabeGadaaakeaacqWGybawdaWgaaWcbaGaemyrauKaeGOmaiZaaSbaaWqaaiabdMgaPbqabaaaleqaaaaa@31A9@, and XE3i
 MathType@MTEF@5@5@+=feaafiart1ev1aaatCvAUfKttLearuWrP9MDH5MBPbIqV92AaeXatLxBI9gBaebbnrfifHhDYfgasaacH8akY=wiFfYdH8Gipec8Eeeu0xXdbba9frFj0=OqFfea0dXdd9vqai=hGuQ8kuc9pgc9s8qqaq=dirpe0xb9q8qiLsFr0=vr0=vr0dc8meaabaqaciaacaGaaeqabaqabeGadaaakeaacqWGybawdaWgaaWcbaGaemyrauKaeG4mamZaaSbaaWqaaiabdMgaPbqabaaaleqaaaaa@31AB@. It is assumed that the product formation for a substrate combination (equation 4a and 5a) is also dependent on the level of the key enzymes (equation 4b and 5b) which control the growth on the respective substrate combinations (equations 2b and 3b).

The enzymes XE1i
 MathType@MTEF@5@5@+=feaafiart1ev1aaatCvAUfKttLearuWrP9MDH5MBPbIqV92AaeXatLxBI9gBaebbnrfifHhDYfgasaacH8akY=wiFfYdH8Gipec8Eeeu0xXdbba9frFj0=OqFfea0dXdd9vqai=hGuQ8kuc9pgc9s8qqaq=dirpe0xb9q8qiLsFr0=vr0=vr0dc8meaabaqaciaacaGaaeqabaqabeGadaaakeaacqWGybawdaWgaaWcbaGaemyrauKaeGymaeZaaSbaaWqaaiabdMgaPbqabaaaleqaaaaa@31A7@, XE2i
 MathType@MTEF@5@5@+=feaafiart1ev1aaatCvAUfKttLearuWrP9MDH5MBPbIqV92AaeXatLxBI9gBaebbnrfifHhDYfgasaacH8akY=wiFfYdH8Gipec8Eeeu0xXdbba9frFj0=OqFfea0dXdd9vqai=hGuQ8kuc9pgc9s8qqaq=dirpe0xb9q8qiLsFr0=vr0=vr0dc8meaabaqaciaacaGaaeqabaqabeGadaaakeaacqWGybawdaWgaaWcbaGaemyrauKaeGOmaiZaaSbaaWqaaiabdMgaPbqabaaaleqaaaaa@31A9@ and *X*_*E*3 _are synthesized from the component of the cell mass X (equation 6a, 7a and 8a) with the rate of synthesis being proportional to the fractional rate r1i*
 MathType@MTEF@5@5@+=feaafiart1ev1aaatCvAUfKttLearuWrP9MDH5MBPbIqV92AaeXatLxBI9gBaebbnrfifHhDYfgasaacH8akY=wiFfYdH8Gipec8Eeeu0xXdbba9frFj0=OqFfea0dXdd9vqai=hGuQ8kuc9pgc9s8qqaq=dirpe0xb9q8qiLsFr0=vr0=vr0dc8meaabaqaciaacaGaaeqabaqabeGadaaakeaacqWGYbGCdaqhaaWcbaGaeGymaeJaemyAaKgabaGaeiOkaOcaaaaa@316D@, r2i*
 MathType@MTEF@5@5@+=feaafiart1ev1aaatCvAUfKttLearuWrP9MDH5MBPbIqV92AaeXatLxBI9gBaebbnrfifHhDYfgasaacH8akY=wiFfYdH8Gipec8Eeeu0xXdbba9frFj0=OqFfea0dXdd9vqai=hGuQ8kuc9pgc9s8qqaq=dirpe0xb9q8qiLsFr0=vr0=vr0dc8meaabaqaciaacaGaaeqabaqabeGadaaakeaacqWGYbGCdaqhaaWcbaGaeGOmaiJaemyAaKgabaGaeiOkaOcaaaaa@316F@ and r3*
 MathType@MTEF@5@5@+=feaafiart1ev1aaatCvAUfKttLearuWrP9MDH5MBPbIqV92AaeXatLxBI9gBaebbnrfifHhDYfgasaacH8akY=wiFfYdH8Gipec8Eeeu0xXdbba9frFj0=OqFfea0dXdd9vqai=hGuQ8kuc9pgc9s8qqaq=dirpe0xb9q8qiLsFr0=vr0=vr0dc8meaabaqaciaacaGaaeqabaqabeGadaaakeaacqWGYbGCdaqhaaWcbaGaeG4mamdabaGaeiOkaOcaaaaa@3016@ at which the organism can potentially grow on the respective substrate combination (equation 6b, 7b and 8b). Nitrogen in the form of *S*_*INS *_is converted to SAAi
 MathType@MTEF@5@5@+=feaafiart1ev1aaatCvAUfKttLearuWrP9MDH5MBPbIqV92AaeXatLxBI9gBaebbnrfifHhDYfgasaacH8akY=wiFfYdH8Gipec8Eeeu0xXdbba9frFj0=OqFfea0dXdd9vqai=hGuQ8kuc9pgc9s8qqaq=dirpe0xb9q8qiLsFr0=vr0=vr0dc8meaabaqaciaacaGaaeqabaqabeGadaaakeaacqWGtbWudaWgaaWcbaGaemyqaeKaemyqae0aaSbaaWqaaiabdMgaPbqabaaaleqaaaaa@31B0@ by a hydrolytic enzyme *X*_*E*4 _(equation 9a and 9b). The fractional rate of growth r1i*
 MathType@MTEF@5@5@+=feaafiart1ev1aaatCvAUfKttLearuWrP9MDH5MBPbIqV92AaeXatLxBI9gBaebbnrfifHhDYfgasaacH8akY=wiFfYdH8Gipec8Eeeu0xXdbba9frFj0=OqFfea0dXdd9vqai=hGuQ8kuc9pgc9s8qqaq=dirpe0xb9q8qiLsFr0=vr0=vr0dc8meaabaqaciaacaGaaeqabaqabeGadaaakeaacqWGYbGCdaqhaaWcbaGaeGymaeJaemyAaKgabaGaeiOkaOcaaaaa@316D@, r2i*
 MathType@MTEF@5@5@+=feaafiart1ev1aaatCvAUfKttLearuWrP9MDH5MBPbIqV92AaeXatLxBI9gBaebbnrfifHhDYfgasaacH8akY=wiFfYdH8Gipec8Eeeu0xXdbba9frFj0=OqFfea0dXdd9vqai=hGuQ8kuc9pgc9s8qqaq=dirpe0xb9q8qiLsFr0=vr0=vr0dc8meaabaqaciaacaGaaeqabaqabeGadaaakeaacqWGYbGCdaqhaaWcbaGaeGOmaiJaemyAaKgabaGaeiOkaOcaaaaa@316F@, r3*
 MathType@MTEF@5@5@+=feaafiart1ev1aaatCvAUfKttLearuWrP9MDH5MBPbIqV92AaeXatLxBI9gBaebbnrfifHhDYfgasaacH8akY=wiFfYdH8Gipec8Eeeu0xXdbba9frFj0=OqFfea0dXdd9vqai=hGuQ8kuc9pgc9s8qqaq=dirpe0xb9q8qiLsFr0=vr0=vr0dc8meaabaqaciaacaGaaeqabaqabeGadaaakeaacqWGYbGCdaqhaaWcbaGaeG4mamdabaGaeiOkaOcaaaaa@3016@are modeled by using a Monod type of kinetics which incorporates a saturation kinetics (KS1i
 MathType@MTEF@5@5@+=feaafiart1ev1aaatCvAUfKttLearuWrP9MDH5MBPbIqV92AaeXatLxBI9gBaebbnrfifHhDYfgasaacH8akY=wiFfYdH8Gipec8Eeeu0xXdbba9frFj0=OqFfea0dXdd9vqai=hGuQ8kuc9pgc9s8qqaq=dirpe0xb9q8qiLsFr0=vr0=vr0dc8meaabaqaciaacaGaaeqabaqabeGadaaakeaacqWGlbWsdaWgaaWcbaGaem4uamLaeGymaeZaaSbaaWqaaiabdMgaPbqabaaaleqaaaaa@31A9@), substrates inhibition (KI1i
 MathType@MTEF@5@5@+=feaafiart1ev1aaatCvAUfKttLearuWrP9MDH5MBPbIqV92AaeXatLxBI9gBaebbnrfifHhDYfgasaacH8akY=wiFfYdH8Gipec8Eeeu0xXdbba9frFj0=OqFfea0dXdd9vqai=hGuQ8kuc9pgc9s8qqaq=dirpe0xb9q8qiLsFr0=vr0=vr0dc8meaabaqaciaacaGaaeqabaqabeGadaaakeaacqWGlbWsdaWgaaWcbaGaemysaKKaeGymaeZaaSbaaWqaaiabdMgaPbqabaaaleqaaaaa@3195@) and nitrogen catabolite repression (*k*_*NT*_) as shown in equation 11, 12 and 13. The enzymes XEki
 MathType@MTEF@5@5@+=feaafiart1ev1aaatCvAUfKttLearuWrP9MDH5MBPbIqV92AaeXatLxBI9gBaebbnrfifHhDYfgasaacH8akY=wiFfYdH8Gipec8Eeeu0xXdbba9frFj0=OqFfea0dXdd9vqai=hGuQ8kuc9pgc9s8qqaq=dirpe0xb9q8qiLsFr0=vr0=vr0dc8meaabaqaciaacaGaaeqabaqabeGadaaakeaacqWGybawdaWgaaWcbaGaemyrauKaem4AaS2aaSbaaWqaaiabdMgaPbqabaaaleqaaaaa@3216@ degrade to form the component of the cell mass X (equation 15a) via a first order degradation kinetics (equation 15b). The mass balance equation for cell mass, product, *S*_*glc*_, SAAi
 MathType@MTEF@5@5@+=feaafiart1ev1aaatCvAUfKttLearuWrP9MDH5MBPbIqV92AaeXatLxBI9gBaebbnrfifHhDYfgasaacH8akY=wiFfYdH8Gipec8Eeeu0xXdbba9frFj0=OqFfea0dXdd9vqai=hGuQ8kuc9pgc9s8qqaq=dirpe0xb9q8qiLsFr0=vr0=vr0dc8meaabaqaciaacaGaaeqabaqabeGadaaakeaacqWGtbWudaWgaaWcbaGaemyqaeKaemyqae0aaSbaaWqaaiabdMgaPbqabaaaleqaaaaa@31B0@, *S*_*amm*_, *S*_*INS *_and enzymes XE1i
 MathType@MTEF@5@5@+=feaafiart1ev1aaatCvAUfKttLearuWrP9MDH5MBPbIqV92AaeXatLxBI9gBaebbnrfifHhDYfgasaacH8akY=wiFfYdH8Gipec8Eeeu0xXdbba9frFj0=OqFfea0dXdd9vqai=hGuQ8kuc9pgc9s8qqaq=dirpe0xb9q8qiLsFr0=vr0=vr0dc8meaabaqaciaacaGaaeqabaqabeGadaaakeaacqWGybawdaWgaaWcbaGaemyrauKaeGymaeZaaSbaaWqaaiabdMgaPbqabaaaleqaaaaa@31A7@, XE2i
 MathType@MTEF@5@5@+=feaafiart1ev1aaatCvAUfKttLearuWrP9MDH5MBPbIqV92AaeXatLxBI9gBaebbnrfifHhDYfgasaacH8akY=wiFfYdH8Gipec8Eeeu0xXdbba9frFj0=OqFfea0dXdd9vqai=hGuQ8kuc9pgc9s8qqaq=dirpe0xb9q8qiLsFr0=vr0=vr0dc8meaabaqaciaacaGaaeqabaqabeGadaaakeaacqWGybawdaWgaaWcbaGaemyrauKaeGOmaiZaaSbaaWqaaiabdMgaPbqabaaaleqaaaaa@31A9@, *X*_*E*3 _and *X*_*E*4 _are shown in equation 16, 17, 20, 21, 22, 23, 24, 25, 26 and 27 respectively.

### Model equations

X(biomass)+Y1,3CO2+Y1,5H2O+Y1,6,iSamm−Y1,1,iSAA,i(amin⁡ o acids)−Y1,4O2=0     (1a)
 MathType@MTEF@5@5@+=feaafiart1ev1aaatCvAUfKttLearuWrP9MDH5MBPbIqV92AaeXatLxBI9gBaebbnrfifHhDYfgasaacH8akY=wiFfYdH8Gipec8Eeeu0xXdbba9frFj0=OqFfea0dXdd9vqai=hGuQ8kuc9pgc9s8qqaq=dirpe0xb9q8qiLsFr0=vr0=vr0dc8meaabaqaciaacaGaaeqabaqabeGadaaakeaadaWfqaqaaiabbIfaybWcbaGaeiikaGIaemOyaiMaemyAaKMaem4Ba8MaemyBa0MaemyyaeMaem4CamNaem4CamNaeiykaKcabeaakiabgUcaRiabbMfaznaaBaaaleaacqaIXaqmcqGGSaalcqaIZaWmaeqaaOGaee4qamKaee4ta80aaSbaaSqaaiabikdaYaqabaGccqGHRaWkcqWGzbqwdaWgaaWcbaGaeGymaeJaeiilaWIaeGynaudabeaakiabdIeainaaBaaaleaacqaIYaGmaeqaaOGaem4ta8Kaey4kaSIaemywaK1aaSbaaSqaaiabigdaXiabcYcaSiabiAda2iabcYcaSiabdMgaPbqabaGccqWGtbWudaWgaaWcbaGaemyyaeMaemyBa0MaemyBa0gabeaakiabgkHiTmaaxababaGaeeywaK1aaSbaaSqaaiabigdaXiabcYcaSiabigdaXiabcYcaSiabbMgaPbqabaGccqWGtbWudaWgaaWcbaGaemyqaeKaemyqaeKaeiilaWIaemyAaKgabeaaaeaacqGGOaakcqWGHbqycyGGTbqBcqGGPbqAcqGGUbGBcqqGGaaicqWGVbWBcqqGGaaicqWGHbqycqWGJbWycqWGPbqAcqWGKbazcqWGZbWCcqGGPaqkaeqaaOGaeyOeI0IaemywaK1aaSbaaSqaaiabigdaXiabcYcaSiabisda0aqabaGccqWGpbWtdaWgaaWcbaGaeGOmaidabeaakiabg2da9iabicdaWiaaxMaacaWLjaWaaeWaaeaacqaIXaqmcqqGHbqyaiaawIcacaGLPaaaaaa@83A2@

μ1,i=μ1,imax⁡ (XE1iXE1iRef)r1i∗      (1b)
 MathType@MTEF@5@5@+=feaafiart1ev1aaatCvAUfKttLearuWrP9MDH5MBPbIqV92AaeXatLxBI9gBaebbnrfifHhDYfgasaacH8akY=wiFfYdH8Gipec8Eeeu0xXdbba9frFj0=OqFfea0dXdd9vqai=hGuQ8kuc9pgc9s8qqaq=dirpe0xb9q8qiLsFr0=vr0=vr0dc8meaabaqaciaacaGaaeqabaqabeGadaaakeaacqaH8oqBdaWgaaWcbaGaeGymaeJaeiilaWIaeeyAaKgabeaakiabg2da9iabeY7aTnaaDaaaleaacqaIXaqmcqGGSaalcqqGPbqAaeaacyGGTbqBcqGGHbqycqGG4baEaaGccqqGGaaidaqadaqaamaalaaabaGaeeiwaG1aaSbaaSqaaiabbweafjabbgdaXmaaBaaameaacqqGPbqAaeqaaaWcbeaaaOqaaiabbIfaynaaDaaaleaacqqGfbqrcqqGXaqmdaWgaaadbaGaeeyAaKgabeaaaSqaaiabbkfasjabbwgaLjabbAgaMbaaaaaakiaawIcacaGLPaaacqqGYbGCdaqhaaWcbaGaeeymaeJaeeyAaKgabaGaey4fIOcaaOGaeeiiaaIaaCzcaiaaxMaadaqadaqaaiabigdaXiabbkgaIbGaayjkaiaawMcaaaaa@56C5@

X + Y_2,3_CO_2 _+ *Y*_2,5_*H*_2_*O *- Y_2,1,*i *_*S*_*AA*,*i *_- Y_2,2 _*S*_*Glc *_- *Y*_2,4_*O*_2 _= 0     (2a)

μ2,i=μ2,imax⁡(XE2iXE2iRe⁡f)r2i∗     (2b)
 MathType@MTEF@5@5@+=feaafiart1ev1aaatCvAUfKttLearuWrP9MDH5MBPbIqV92AaeXatLxBI9gBaebbnrfifHhDYfgasaacH8akY=wiFfYdH8Gipec8Eeeu0xXdbba9frFj0=OqFfea0dXdd9vqai=hGuQ8kuc9pgc9s8qqaq=dirpe0xb9q8qiLsFr0=vr0=vr0dc8meaabaqaciaacaGaaeqabaqabeGadaaakeaacqaH8oqBdaWgaaWcbaGaeGOmaiJaeiilaWIaeeyAaKgabeaakiabg2da9iabeY7aTnaaDaaaleaacqaIYaGmcqGGSaalcqqGPbqAaeaacyGGTbqBcqGGHbqycqGG4baEaaGcdaqadaqaamaalaaabaGaemiwaG1aaSbaaSqaaiabdweafjabikdaYmaaBaaameaacqWGPbqAaeqaaaWcbeaaaOqaaiabdIfaynaaDaaaleaacqWGfbqrcqaIYaGmdaWgaaadbaGaemyAaKgabeaaaSqaaiGbckfasjabcwgaLjabdAgaMbaaaaaakiaawIcacaGLPaaacqWGYbGCdaqhaaWcbaGaeGOmaiJaemyAaKgabaGaey4fIOcaaOGaaCzcaiaaxMaadaqadaqaaiabikdaYiabbkgaIbGaayjkaiaawMcaaaaa@556E@

X + Y_3,3_CO_2 _+ *Y*_3,5_*H*_2_*O *- Y_3,2 _*S*_*Glc *_- *Y*_3,4_*O*_2 _- *Y*_3,6_*S*_*amm *_= 0     (3a)

μ3=μ3max⁡(XE3XE3Re⁡f)r3∗     (3b)
 MathType@MTEF@5@5@+=feaafiart1ev1aaatCvAUfKttLearuWrP9MDH5MBPbIqV92AaeXatLxBI9gBaebbnrfifHhDYfgasaacH8akY=wiFfYdH8Gipec8Eeeu0xXdbba9frFj0=OqFfea0dXdd9vqai=hGuQ8kuc9pgc9s8qqaq=dirpe0xb9q8qiLsFr0=vr0=vr0dc8meaabaqaciaacaGaaeqabaqabeGadaaakeaacqaH8oqBdaWgaaWcbaGaee4mamdabeaakiabg2da9iabeY7aTnaaDaaaleaacqaIZaWmaeaacyGGTbqBcqGGHbqycqGG4baEaaGcdaqadaqaamaalaaabaGaemiwaG1aaSbaaSqaaiabdweafjabiodaZaqabaaakeaacqWGybawdaqhaaWcbaGaemyrauKaeG4mamdabaGagiOuaiLaeiyzauMaemOzaygaaaaaaOGaayjkaiaawMcaaiabdkhaYnaaDaaaleaacqaIZaWmaeaacqGHxiIkaaGccaWLjaGaaCzcamaabmaabaGaeG4mamJaeeOyaigacaGLOaGaayzkaaaaaa@4C80@

P + Y_4,3_CO_2 _+ *Y*_4,5_*H*_2_*O *- Y_4,1i _*S*_*AA*,*i *_- Y_4,2 _*S*_*Glc *_- *Y*_4,4_*O*_2 _= 0     (4a)

qp2,i=qp2,imax⁡(XE2iXE2iRe⁡f)r2P,i∗     (4b)
 MathType@MTEF@5@5@+=feaafiart1ev1aaatCvAUfKttLearuWrP9MDH5MBPbIqV92AaeXatLxBI9gBaebbnrfifHhDYfgasaacH8akY=wiFfYdH8Gipec8Eeeu0xXdbba9frFj0=OqFfea0dXdd9vqai=hGuQ8kuc9pgc9s8qqaq=dirpe0xb9q8qiLsFr0=vr0=vr0dc8meaabaqaciaacaGaaeqabaqabeGadaaakeaacqWGXbqCdaWgaaWcbaGaemiCaaNaeGOmaiJaeiilaWIaemyAaKgabeaakiabg2da9iabdghaXnaaDaaaleaacqWGWbaCcqaIYaGmcqGGSaalcqWGPbqAaeaacyGGTbqBcqGGHbqycqGG4baEaaGcdaqadaqaamaalaaabaGaemiwaG1aaSbaaSqaaiabdweafjabikdaYmaaBaaameaacqWGPbqAaeqaaaWcbeaaaOqaaiabdIfaynaaDaaaleaacqWGfbqrcqaIYaGmdaWgaaadbaGaemyAaKgabeaaaSqaaiGbckfasjabcwgaLjabdAgaMbaaaaaakiaawIcacaGLPaaacqWGYbGCdaqhaaWcbaGaeGOmaiJaemiuaaLaeiilaWIaemyAaKgabaGaey4fIOcaaOGaaCzcaiaaxMaadaqadaqaaiabisda0iabbkgaIbGaayjkaiaawMcaaaaa@59BB@

P + Y_5,3_CO_2 _+ *Y*_5,5_*H*_2_*O *- Y_5,6 _*S*_*amm *_- Y_5,2 _*S*_*Glc *_- *Y*_5,4_*O*_2 _= 0     (5a)

qp3=qp3max⁡(XE3XE3Re⁡f)r3∗     (5b)
 MathType@MTEF@5@5@+=feaafiart1ev1aaatCvAUfKttLearuWrP9MDH5MBPbIqV92AaeXatLxBI9gBaebbnrfifHhDYfgasaacH8akY=wiFfYdH8Gipec8Eeeu0xXdbba9frFj0=OqFfea0dXdd9vqai=hGuQ8kuc9pgc9s8qqaq=dirpe0xb9q8qiLsFr0=vr0=vr0dc8meaabaqaciaacaGaaeqabaqabeGadaaakeaacqWGXbqCdaWgaaWcbaGaemiCaaNaeG4mamdabeaakiabg2da9iabdghaXnaaDaaaleaacqWGWbaCcqaIZaWmaeaacyGGTbqBcqGGHbqycqGG4baEaaGcdaqadaqaamaalaaabaGaemiwaG1aaSbaaSqaaiabdweafjabiodaZaqabaaakeaacqWGybawdaqhaaWcbaGaemyrauKaeG4mamdabaGagiOuaiLaeiyzauMaemOzaygaaaaaaOGaayjkaiaawMcaaiabdkhaYnaaDaaaleaacqaIZaWmaeaacqGHxiIkaaGccaWLjaGaaCzcamaabmaabaGaeGynauJaeeOyaigacaGLOaGaayzkaaaaaa@4EC7@

XE1i−X=0     (6a)
 MathType@MTEF@5@5@+=feaafiart1ev1aaatCvAUfKttLearuWrP9MDH5MBPbIqV92AaeXatLxBI9gBaebbnrfifHhDYfgasaacH8akY=wiFfYdH8Gipec8Eeeu0xXdbba9frFj0=OqFfea0dXdd9vqai=hGuQ8kuc9pgc9s8qqaq=dirpe0xb9q8qiLsFr0=vr0=vr0dc8meaabaqaciaacaGaaeqabaqabeGadaaakeaacqqGybawdaWgaaWcbaGaeeyrauKaeeymaeZaaSbaaWqaaiabbMgaPbqabaaaleqaaOGaeyOeI0IaemiwaGLaeyypa0JaeGimaaJaaCzcaiaaxMaadaqadaqaaiabiAda2iabbggaHbGaayjkaiaawMcaaaaa@3ACE@

rE1i=kE1ir1i∗     (6b)
 MathType@MTEF@5@5@+=feaafiart1ev1aaatCvAUfKttLearuWrP9MDH5MBPbIqV92AaeXatLxBI9gBaebbnrfifHhDYfgasaacH8akY=wiFfYdH8Gipec8Eeeu0xXdbba9frFj0=OqFfea0dXdd9vqai=hGuQ8kuc9pgc9s8qqaq=dirpe0xb9q8qiLsFr0=vr0=vr0dc8meaabaqaciaacaGaaeqabaqabeGadaaakeaacqqGYbGCdaWgaaWcbaGaeeyrauKaeGymaeZaaSbaaWqaaiabbMgaPbqabaaaleqaaOGaeyypa0Jaem4AaS2aaSbaaSqaaiabdweafjabigdaXmaaBaaameaacqWGPbqAaeqaaaWcbeaakiabdkhaYnaaDaaaleaacqaIXaqmcqWGPbqAaeaacqGHxiIkaaGccaWLjaGaaCzcamaabmaabaGaeGOnayJaeeOyaigacaGLOaGaayzkaaaaaa@4200@

XE2i−X=0     (7a)
 MathType@MTEF@5@5@+=feaafiart1ev1aaatCvAUfKttLearuWrP9MDH5MBPbIqV92AaeXatLxBI9gBaebbnrfifHhDYfgasaacH8akY=wiFfYdH8Gipec8Eeeu0xXdbba9frFj0=OqFfea0dXdd9vqai=hGuQ8kuc9pgc9s8qqaq=dirpe0xb9q8qiLsFr0=vr0=vr0dc8meaabaqaciaacaGaaeqabaqabeGadaaakeaacqqGybawdaWgaaWcbaGaeeyrauKaeGOmaiZaaSbaaWqaaiabbMgaPbqabaaaleqaaOGaeyOeI0IaemiwaGLaeyypa0JaeGimaaJaaCzcaiaaxMaadaqadaqaaiabiEda3iabbggaHbGaayjkaiaawMcaaaaa@3AD9@

rE2i=kE2ir2i*     (7b)
 MathType@MTEF@5@5@+=feaafiart1ev1aaatCvAUfKttLearuWrP9MDH5MBPbIqV92AaeXatLxBI9gBaebbnrfifHhDYfgasaacH8akY=wiFfYdH8Gipec8Eeeu0xXdbba9frFj0=OqFfea0dXdd9vqai=hGuQ8kuc9pgc9s8qqaq=dirpe0xb9q8qiLsFr0=vr0=vr0dc8meaabaqaciaacaGaaeqabaqabeGadaaakeaacqqGYbGCdaWgaaWcbaGaeeyrauKaeeOmaiZaaSbaaWqaaiabbMgaPbqabaaaleqaaOGaeyypa0Jaem4AaS2aaSbaaSqaaiabdweafjabikdaYmaaBaaameaacqWGPbqAaeqaaaWcbeaakiabdkhaYnaaDaaaleaacqaIYaGmcqWGPbqAaeaacqGGQaGkaaGccaWLjaGaaCzcamaabmaabaGaeG4naCJaeeOyaigacaGLOaGaayzkaaaaaa@41EE@

X_E3 _- *X *= 0     (8a)

rE3=kE3r3*     (8b)
 MathType@MTEF@5@5@+=feaafiart1ev1aaatCvAUfKttLearuWrP9MDH5MBPbIqV92AaeXatLxBI9gBaebbnrfifHhDYfgasaacH8akY=wiFfYdH8Gipec8Eeeu0xXdbba9frFj0=OqFfea0dXdd9vqai=hGuQ8kuc9pgc9s8qqaq=dirpe0xb9q8qiLsFr0=vr0=vr0dc8meaabaqaciaacaGaaeqabaqabeGadaaakeaacqqGYbGCdaWgaaWcbaGaeeyrauKaee4mamdabeaakiabg2da9iabdUgaRnaaBaaaleaacqWGfbqrcqaIZaWmaeqaaOGaemOCai3aa0baaSqaaiabiodaZaqaaiabcQcaQaaakiaaxMaacaWLjaWaaeWaaeaacqaI4aaocqqGIbGyaiaawIcacaGLPaaaaaa@3D77@

−SINS+∑i=120SAA,iφi=0     (9a)
 MathType@MTEF@5@5@+=feaafiart1ev1aaatCvAUfKttLearuWrP9MDH5MBPbIqV92AaeXatLxBI9gBaebbnrfifHhDYfgasaacH8akY=wiFfYdH8Gipec8Eeeu0xXdbba9frFj0=OqFfea0dXdd9vqai=hGuQ8kuc9pgc9s8qqaq=dirpe0xb9q8qiLsFr0=vr0=vr0dc8meaabaqaciaacaGaaeqabaqabeGadaaakeaacqGHsislcqWGtbWudaWgaaWcbaGaemysaKKaemOta4Kaem4uamfabeaakiabgUcaRmaaqahabaGaem4uam1aaSbaaSqaaiabdgeabjabdgeabjabcYcaSiabdMgaPbqabaacciGccqWFgpGzdaWgaaWcbaGaemyAaKgabeaaaeaacqWGPbqAcqGH9aqpcqaIXaqmaeaacqaIYaGmcqaIWaama0GaeyyeIuoakiabg2da9iabicdaWiaaxMaacaWLjaWaaeWaaeaacqaI5aqocqqGHbqyaiaawIcacaGLPaaaaaa@4AC8@

rdiss=(XE4XE4Re⁡f)K4r4*     (9b)
 MathType@MTEF@5@5@+=feaafiart1ev1aaatCvAUfKttLearuWrP9MDH5MBPbIqV92AaeXatLxBI9gBaebbnrfifHhDYfgasaacH8akY=wiFfYdH8Gipec8Eeeu0xXdbba9frFj0=OqFfea0dXdd9vqai=hGuQ8kuc9pgc9s8qqaq=dirpe0xb9q8qiLsFr0=vr0=vr0dc8meaabaqaciaacaGaaeqabaqabeGadaaakeaacqqGYbGCdaWgaaWcbaGaeeizaqMaeeyAaKMaee4CamNaee4Camhabeaakiabg2da9maabmaabaWaaSaaaeaacqWGybawdaWgaaWcbaGaemyrauKaeGinaqdabeaaaOqaaiabdIfaynaaDaaaleaacqWGfbqrcqaI0aanaeaacyGGsbGucqGGLbqzcqWGMbGzaaaaaaGccaGLOaGaayzkaaGaem4saS0aaSbaaSqaaiabisda0aqabaGccqWGYbGCdaqhaaWcbaGaeGinaqdabaGaeiOkaOcaaOGaaCzcaiaaxMaadaqadaqaaiabiMda5iabbkgaIbGaayjkaiaawMcaaaaa@4C0D@

φi=SAA,iat time t=0,∑i=120SAA,iat time t=0,
 MathType@MTEF@5@5@+=feaafiart1ev1aaatCvAUfKttLearuWrP9MDH5MBPbIqV92AaeXatLxBI9gBaebbnrfifHhDYfgasaacH8akY=wiFfYdH8Gipec8Eeeu0xXdbba9frFj0=OqFfea0dXdd9vqai=hGuQ8kuc9pgc9s8qqaq=dirpe0xb9q8qiLsFr0=vr0=vr0dc8meaabaqaciaacaGaaeqabaqabeGadaaakeaaiiGacqWFgpGzdaWgaaWcbaGaemyAaKgabeaakiabg2da9maalaaabaGaem4uam1aaSbaaSqaaiabdgeabjabdgeabjabcYcaSiabdMgaPbqabaGccqWGHbqycqWG0baDcqqGGaaicqWG0baDcqWGPbqAcqWGTbqBcqWGLbqzcqqGGaaicqWG0baDcqGH9aqpcqaIWaamcqGGSaalaeaadaaeWbqaaiabdofatnaaBaaaleaacqWGbbqqcqWGbbqqcqGGSaalcqWGPbqAaeqaaOGaemyyaeMaemiDaqNaeeiiaaIaemiDaqNaemyAaKMaemyBa0MaemyzauMaeeiiaaIaemiDaqNaeyypa0JaeGimaaJaeiilaWcaleaacqWGPbqAcqGH9aqpcqaIXaqmaeaacqaIYaGmcqaIWaama0GaeyyeIuoaaaaaaa@6014@;distribution coefficients     (10)

r1i∗=SAA,iKs1,i+SAA,i+NT2kNT1,i+SAA,i2KI1,i     (11)
 MathType@MTEF@5@5@+=feaafiart1ev1aaatCvAUfKttLearuWrP9MDH5MBPbIqV92AaeXatLxBI9gBaebbnrfifHhDYfgasaacH8akY=wiFfYdH8Gipec8Eeeu0xXdbba9frFj0=OqFfea0dXdd9vqai=hGuQ8kuc9pgc9s8qqaq=dirpe0xb9q8qiLsFr0=vr0=vr0dc8meaabaqaciaacaGaaeqabaqabeGadaaakeaacqqGYbGCdaqhaaWcbaGaeGymaeJaeeyAaKgabaGaey4fIOcaaOGaeyypa0ZaaSaaaeaacqqGtbWudaWgaaWcbaGaeeyqaeKaeeyqaeKaeeilaWIaeeyAaKgabeaaaOqaaiabbUealjabbohaZnaaBaaaleaacqaIXaqmcqGGSaalieGacqWFPbqAaeqaaOGaey4kaSIaee4uam1aaSbaaSqaaiabbgeabjabbgeabjabbYcaSiabbMgaPbqabaGccqGHRaWkdaWcaaqaaiabd6eaonaaDaaaleaacqWGubavaeaacqaIYaGmaaaakeaacqWGRbWAdaWgaaWcbaGaemOta4KaemivaqLaeGymaeJaeiilaWIaemyAaKgabeaaaaGccqGHRaWkdaWcaaqaaiabbofatnaaDaaaleaacqqGbbqqcqqGbbqqcqqGSaalcqqGPbqAaeaacqaIYaGmaaaakeaacqqGlbWsdaWgaaWcbaGaeeysaKKaeGymaeJaeiilaWIaeeyAaKgabeaaaaaaaOGaaCzcaiaaxMaadaqadaqaceaaxRUaeGymaeJaeGymaedacaGLOaGaayzkaaaaaa@63E9@

r2i∗=SAA,iKs2,i+SAA,i+NT2kNT2,i+SAA,i2KI2,iSglcKs2,2+Sglc+SGlc2KI2,2     (12)
 MathType@MTEF@5@5@+=feaafiart1ev1aaatCvAUfKttLearuWrP9MDH5MBPbIqV92AaeXatLxBI9gBaebbnrfifHhDYfgasaacH8akY=wiFfYdH8Gipec8Eeeu0xXdbba9frFj0=OqFfea0dXdd9vqai=hGuQ8kuc9pgc9s8qqaq=dirpe0xb9q8qiLsFr0=vr0=vr0dc8meaabaqaciaacaGaaeqabaqabeGadaaakeaacqqGYbGCdaqhaaWcbaGaeGOmaiJaeeyAaKgabaGaey4fIOcaaOGaeyypa0ZaaSaaaeaacqqGtbWudaWgaaWcbaGaeeyqaeKaeeyqaeKaeeilaWIaeeyAaKgabeaaaOqaaiabbUealjabbohaZnaaBaaaleaacqaIYaGmcqGGSaalieGacqWFPbqAaeqaaOGaey4kaSIaee4uam1aaSbaaSqaaiabbgeabjabbgeabjabbYcaSiabbMgaPbqabaGccqGHRaWkdaWcaaqaaiabd6eaonaaDaaaleaacqWGubavaeaacqaIYaGmaaaakeaacqWGRbWAdaWgaaWcbaGaemOta4KaemivaqLaeGOmaiJaeiilaWIaemyAaKgabeaaaaGccqGHRaWkdaWcaaqaaiabbofatnaaDaaaleaacqqGbbqqcqqGbbqqcqqGSaalcqqGPbqAaeaacqaIYaGmaaaakeaacqqGlbWsdaWgaaWcbaGaeeysaKKaeeOmaiJaeiilaWIaeeyAaKgabeaaaaaaaOWaaSaaaeaacqqGtbWudaWgaaWcbaGaee4zaCMaeeiBaWMaee4yamgabeaaaOqaaiabbUealjabbohaZnaaBaaaleaacqaIYaGmcqGGSaalcqaIYaGmaeqaaOGaey4kaSIaee4uam1aaSbaaSqaaiabbEgaNjabbYgaSjabbogaJbqabaGccqGHRaWkdaWcaaqaaiabbofatnaaDaaaleaacqqGhbWrcqqGSbaBcqqGJbWyaeaacqaIYaGmaaaakeaacqqGlbWsdaWgaaWcbaGaeeysaKKaeGOmaiJaeiilaWIaeGOmaidabeaaaaaaaOGaaCzcaiaaxMaadaqadaqaceaaxRUaeGymaeJaeGOmaidacaGLOaGaayzkaaaaaa@8163@

r3∗=SammKs3,3+Samm+Samm2KI3,3SglcKs3,2+Sglc+SGlc2KI3,2     (13)
 MathType@MTEF@5@5@+=feaafiart1ev1aaatCvAUfKttLearuWrP9MDH5MBPbIqV92AaeXatLxBI9gBaebbnrfifHhDYfgasaacH8akY=wiFfYdH8Gipec8Eeeu0xXdbba9frFj0=OqFfea0dXdd9vqai=hGuQ8kuc9pgc9s8qqaq=dirpe0xb9q8qiLsFr0=vr0=vr0dc8meaabaqaciaacaGaaeqabaqabeGadaaakeaacqqGYbGCdaqhaaWcbaGaee4mamdabaGaey4fIOcaaOGaeyypa0ZaaSaaaeaacqqGtbWudaWgaaWcbaGaeeyyaeMaeeyBa0MaeeyBa0gabeaaaOqaaiabbUealjabbohaZnaaBaaaleaacqaIZaWmcqGGSaalcqaIZaWmaeqaaOGaey4kaSIaee4uam1aaSbaaSqaaiabbggaHjabb2gaTjabb2gaTbqabaGccqGHRaWkdaWcaaqaaiabbofatnaaDaaaleaacqqGHbqycqqGTbqBcqqGTbqBaeaacqaIYaGmaaaakeaacqqGlbWsdaWgaaWcbaGaeeysaKKaee4mamJaeiilaWIaeG4mamdabeaaaaaaaOWaaSaaaeaacqqGtbWudaWgaaWcbaGaee4zaCMaeeiBaWMaee4yamgabeaaaOqaaiabbUealjabbohaZnaaBaaaleaacqaIZaWmcqGGSaalcqaIYaGmaeqaaOGaey4kaSIaee4uam1aaSbaaSqaaiabbEgaNjabbYgaSjabbogaJbqabaGccqGHRaWkdaWcaaqaaiabbofatnaaDaaaleaacqqGhbWrcqqGSbaBcqqGJbWyaeaacqaIYaGmaaaakeaacqqGlbWsdaWgaaWcbaGaeeysaKKaee4mamJaeiilaWIaeGOmaidabeaaaaaaaOGaaCzcaiaaxMaadaqadaqaceaaxRUaeGymaeJaeG4mamdacaGLOaGaayzkaaaaaa@72EF@

r4*=(SINSSINS+KS4)     (14)
 MathType@MTEF@5@5@+=feaafiart1ev1aaatCvAUfKttLearuWrP9MDH5MBPbIqV92AaeXatLxBI9gBaebbnrfifHhDYfgasaacH8akY=wiFfYdH8Gipec8Eeeu0xXdbba9frFj0=OqFfea0dXdd9vqai=hGuQ8kuc9pgc9s8qqaq=dirpe0xb9q8qiLsFr0=vr0=vr0dc8meaabaqaciaacaGaaeqabaqabeGadaaakeaacqWGYbGCdaqhaaWcbaGaeGinaqdabaGaeiOkaOcaaOGaeyypa0ZaaeWaaeaadaWcaaqaaiabdofatnaaBaaaleaacqWGjbqscqWGobGtcqWGtbWuaeqaaaGcbaGaem4uam1aaSbaaSqaaiabdMeajjabd6eaojabdofatbqabaGccqGHRaWkcqWGlbWsdaWgaaWcbaGaem4uamLaeGinaqdabeaaaaaakiaawIcacaGLPaaacaWLjaGaaCzcamaabmaabaGaeGymaeJaeGinaqdacaGLOaGaayzkaaaaaa@4578@

XEki−X=0     (15a)
 MathType@MTEF@5@5@+=feaafiart1ev1aaatCvAUfKttLearuWrP9MDH5MBPbIqV92AaeXatLxBI9gBaebbnrfifHhDYfgasaacH8akY=wiFfYdH8Gipec8Eeeu0xXdbba9frFj0=OqFfea0dXdd9vqai=hGuQ8kuc9pgc9s8qqaq=dirpe0xb9q8qiLsFr0=vr0=vr0dc8meaabaqaciaacaGaaeqabaqabeGadaaakeaacqWGybawdaWgaaWcbaGaemyrauKaem4AaS2aaSbaaWqaaiabdMgaPbqabaaaleqaaOGaeyOeI0IaemiwaGLaeyypa0JaeGimaaJaaCzcaiaaxMaadaqadaqaaiabigdaXiabiwda1iabbggaHbGaayjkaiaawMcaaaaa@3C38@

rdegi=BetaK,iXEki     (15b)
 MathType@MTEF@5@5@+=feaafiart1ev1aaatCvAUfKttLearuWrP9MDH5MBPbIqV92AaeXatLxBI9gBaebbnrfifHhDYfgasaacH8akY=wiFfYdH8Gipec8Eeeu0xXdbba9frFj0=OqFfea0dXdd9vqai=hGuQ8kuc9pgc9s8qqaq=dirpe0xb9q8qiLsFr0=vr0=vr0dc8meaabaqaciaacaGaaeqabaqabeGadaaakeaacqqGYbGCdaWgaaWcbaGaeeizaqMaeeyzauMaee4zaC2aaSbaaWqaaiabbMgaPbqabaaaleqaaOGaeyypa0JaemOqaiKaemyzauMaemiDaqNaemyyae2aaSbaaSqaaiabdUealjabcYcaSiabdMgaPbqabaGccqqGybawdaWgaaWcbaGaeeyrauKaee4AaS2aaSbaaWqaaiabbMgaPbqabaaaleqaaOGaaCzcaiaaxMaadaqadaqaaiabigdaXiabiwda1iabbkgaIbGaayjkaiaawMcaaaaa@48F1@

dXdt=μ X     (16)
 MathType@MTEF@5@5@+=feaafiart1ev1aaatCvAUfKttLearuWrP9MDH5MBPbIqV92AaeXatLxBI9gBaebbnrfifHhDYfgasaacH8akY=wiFfYdH8Gipec8Eeeu0xXdbba9frFj0=OqFfea0dXdd9vqai=hGuQ8kuc9pgc9s8qqaq=dirpe0xb9q8qiLsFr0=vr0=vr0dc8meaabaqaciaacaGaaeqabaqabeGadaaakeaadaWcaaqaaiabbsgaKjabbIfaybqaaiabbsgaKjabbsha0baacqGH9aqpcqaH8oqBcqqGGaaicqqGybawcaWLjaGaaCzcamaabmaabaGaeeymaeJaeeOnaydacaGLOaGaayzkaaaaaa@3B63@

dPdt=qPX     (17)
 MathType@MTEF@5@5@+=feaafiart1ev1aaatCvAUfKttLearuWrP9MDH5MBPbIqV92AaeXatLxBI9gBaebbnrfifHhDYfgasaacH8akY=wiFfYdH8Gipec8Eeeu0xXdbba9frFj0=OqFfea0dXdd9vqai=hGuQ8kuc9pgc9s8qqaq=dirpe0xb9q8qiLsFr0=vr0=vr0dc8meaabaqaciaacaGaaeqabaqabeGadaaakeaadaWcaaqaaiabbsgaKjabbcfaqbqaaiabbsgaKjabbsha0baacqGH9aqpcqqGXbqCdaWgaaWcbaGaeeiuaafabeaakiabbIfayjaaxMaacaWLjaWaaeWaaeaacqqGXaqmcqqG3aWnaiaawIcacaGLPaaaaaa@3B9E@

Where *μ *is the overall specific growth rate, which is the sum of the fraction of specific growth rates on individual substrate combination and is given by the equation 18.

μ=∑i=120μ1i+∑120μ2i+μ3     (18)
 MathType@MTEF@5@5@+=feaafiart1ev1aaatCvAUfKttLearuWrP9MDH5MBPbIqV92AaeXatLxBI9gBaebbnrfifHhDYfgasaacH8akY=wiFfYdH8Gipec8Eeeu0xXdbba9frFj0=OqFfea0dXdd9vqai=hGuQ8kuc9pgc9s8qqaq=dirpe0xb9q8qiLsFr0=vr0=vr0dc8meaabaqaciaacaGaaeqabaqabeGadaaakeaacqaH8oqBcqGH9aqpdaaeWbqaaiabeY7aTnaaBaaaleaacqqGXaqmcqqGPbqAaeqaaaqaaiabbMgaPjabg2da9iabbgdaXaqaaiabbkdaYiabbcdaWaqdcqGHris5aOGaey4kaSYaaabCaeaacqaH8oqBdaWgaaWcbaGaeGOmaiJaemyAaKgabeaaaeaacqaIXaqmaeaacqaIYaGmcqaIWaama0GaeyyeIuoakiabgUcaRiabeY7aTnaaBaaaleaacqaIZaWmaeqaaOGaaCzcaiaaxMaadaqadaqaaiabigdaXiabiIda4aGaayjkaiaawMcaaaaa@4D86@

Likewise, the overall specific product formation rate (q_p_) is given by equation 19.

qP=∑i=120qP2,i+qp3     (19)
 MathType@MTEF@5@5@+=feaafiart1ev1aaatCvAUfKttLearuWrP9MDH5MBPbIqV92AaeXatLxBI9gBaebbnrfifHhDYfgasaacH8akY=wiFfYdH8Gipec8Eeeu0xXdbba9frFj0=OqFfea0dXdd9vqai=hGuQ8kuc9pgc9s8qqaq=dirpe0xb9q8qiLsFr0=vr0=vr0dc8meaabaqaciaacaGaaeqabaqabeGadaaakeaacqWGXbqCdaWgaaWcbaGaemiuaafabeaakiabg2da9maaqahabaGaemyCae3aaSbaaSqaaiabdcfaqjabikdaYiabcYcaSiabdMgaPbqabaaabaGaemyAaKMaeyypa0JaeGymaedabaGaeGOmaiJaeGimaadaniabggHiLdGccqGHRaWkcqWGXbqCdaWgaaWcbaGaemiCaaNaeG4mamdabeaakiaaxMaacaWLjaWaaeWaaeaacqaIXaqmcqaI5aqoaiaawIcacaGLPaaaaaa@4778@

dSglcdt=−[∑i=120Y2,2μ2,i+Y3,2μ3+∑i=120Y4,2qp2,i+Y5,2qp,3]X     (20)
 MathType@MTEF@5@5@+=feaafiart1ev1aaatCvAUfKttLearuWrP9MDH5MBPbIqV92AaeXatLxBI9gBaebbnrfifHhDYfgasaacH8akY=wiFfYdH8Gipec8Eeeu0xXdbba9frFj0=OqFfea0dXdd9vqai=hGuQ8kuc9pgc9s8qqaq=dirpe0xb9q8qiLsFr0=vr0=vr0dc8meaabaqaciaacaGaaeqabaqabeGadaaakeaadaWcaaqaaiabdsgaKjabdofatnaaBaaaleaacqWGNbWzcqWGSbaBcqWGJbWyaeqaaaGcbaGaemizaqMaemiDaqhaaiabg2da9iabgkHiTmaadmaabaWaaabCaeaacqWGzbqwdaWgaaWcbaGaeGOmaiJaeiilaWIaeGOmaidabeaakiabeY7aTnaaBaaaleaacqaIYaGmcqGGSaalcqWGPbqAaeqaaaqaaiabdMgaPjabg2da9iabigdaXaqaaiabikdaYiabicdaWaqdcqGHris5aOGaey4kaSIaemywaK1aaSbaaSqaaiabiodaZiabcYcaSiabikdaYaqabaGccqaH8oqBdaWgaaWcbaGaeG4mamdabeaakiabgUcaRmaaqahabaGaemywaK1aaSbaaSqaaiabisda0iabcYcaSiabikdaYaqabaGccqWGXbqCdaWgaaWcbaGaemiCaaNaeGOmaiJaeiilaWIaemyAaKgabeaaaeaacqWGPbqAcqGH9aqpcqaIXaqmaeaacqaIYaGmcqaIWaama0GaeyyeIuoakiabgUcaRiabdMfaznaaBaaaleaacqaI1aqncqGGSaalcqaIYaGmaeqaaOGaemyCae3aaSbaaSqaaiabdchaWjabcYcaSiabiodaZaqabaaakiaawUfacaGLDbaacqWGybawcaWLjaGaaCzcamaabmaabaGaeGOmaiJaeGimaadacaGLOaGaayzkaaaaaa@756A@

dSAAidt=K4(XE4XE4Re⁡f)(SINSSINS+KS4)φiX−[Y1,1,iμ1,i+Y2,1,iμ2,i+Y4,1,iqp,2,i]X     (21)
 MathType@MTEF@5@5@+=feaafiart1ev1aaatCvAUfKttLearuWrP9MDH5MBPbIqV92AaeXatLxBI9gBaebbnrfifHhDYfgasaacH8akY=wiFfYdH8Gipec8Eeeu0xXdbba9frFj0=OqFfea0dXdd9vqai=hGuQ8kuc9pgc9s8qqaq=dirpe0xb9q8qiLsFr0=vr0=vr0dc8meaabaqaciaacaGaaeqabaqabeGadaaakeaadaWcaaqaaiabdsgaKjabdofatnaaBaaaleaacqWGbbqqcqWGbbqqcqWGPbqAaeqaaaGcbaGaemizaqMaemiDaqhaaiabg2da9iabdUealnaaBaaaleaacqaI0aanaeqaaOWaaeWaaeaadaWcaaqaaiabdIfaynaaBaaaleaacqWGfbqrcqaI0aanaeqaaaGcbaGaemiwaG1aa0baaSqaaiabdweafjabisda0aqaaiGbckfasjabcwgaLjabdAgaMbaaaaaakiaawIcacaGLPaaadaqadaqaamaalaaabaGaem4uam1aaSbaaSqaaiabdMeajjabd6eaojabdofatbqabaaakeaacqWGtbWudaWgaaWcbaGaemysaKKaemOta4Kaem4uamfabeaakiabgUcaRiabdUealnaaBaaaleaacqWGtbWucqaI0aanaeqaaaaaaOGaayjkaiaawMcaaGGaciab=z8aMnaaBaaaleaacqWGPbqAaeqaaOGaemiwaGLaeyOeI0YaamWaaeaacqWGzbqwdaWgaaWcbaGaeGymaeJaeiilaWIaeGymaeJaeiilaWIaemyAaKgabeaakiabeY7aTnaaBaaaleaacqaIXaqmcqGGSaalcqWGPbqAaeqaaOGaey4kaSIaemywaK1aaSbaaSqaaiabikdaYiabcYcaSiabigdaXiabcYcaSiabdMgaPbqabaGccqaH8oqBdaWgaaWcbaGaeGOmaiJaeiilaWIaemyAaKgabeaakiabgUcaRiabdMfaznaaBaaaleaacqaI0aancqGGSaalcqaIXaqmcqGGSaalcqWGPbqAaeqaaOGaemyCae3aaSbaaSqaaiabdchaWjabcYcaSiabikdaYiabcYcaSiabdMgaPbqabaaakiaawUfacaGLDbaacqWGybawcaWLjaGaaCzcamaabmaabaGaeGOmaiJaeGymaedacaGLOaGaayzkaaaaaa@889A@

dSammdt=[∑i=120Y1,6,iμ1,i−Y3,6μ3−Y5,6qp,3]X     (22)
 MathType@MTEF@5@5@+=feaafiart1ev1aaatCvAUfKttLearuWrP9MDH5MBPbIqV92AaeXatLxBI9gBaebbnrfifHhDYfgasaacH8akY=wiFfYdH8Gipec8Eeeu0xXdbba9frFj0=OqFfea0dXdd9vqai=hGuQ8kuc9pgc9s8qqaq=dirpe0xb9q8qiLsFr0=vr0=vr0dc8meaabaqaciaacaGaaeqabaqabeGadaaakeaadaWcaaqaaiabdsgaKjabdofatnaaBaaaleaacqWGHbqycqWGTbqBcqWGTbqBaeqaaaGcbaGaemizaqMaemiDaqhaaiabg2da9maadmaabaWaaabCaeaacqWGzbqwdaWgaaWcbaGaeGymaeJaeiilaWIaeGOnayJaeiilaWIaemyAaKgabeaakiabeY7aTnaaBaaaleaacqaIXaqmcqGGSaalcqWGPbqAaeqaaaqaaiabdMgaPjabg2da9iabigdaXaqaaiabikdaYiabicdaWaqdcqGHris5aOGaeyOeI0IaemywaK1aaSbaaSqaaiabiodaZiabcYcaSiabiAda2aqabaGccqaH8oqBdaWgaaWcbaGaeG4mamdabeaakiabgkHiTiabdMfaznaaBaaaleaacqaI1aqncqGGSaalcqaI2aGnaeqaaOGaemyCae3aaSbaaSqaaiabdchaWjabcYcaSiabiodaZaqabaaakiaawUfacaGLDbaacqWGybawcaWLjaGaaCzcamaabmaabaGaeGOmaiJaeGOmaidacaGLOaGaayzkaaaaaa@6436@

dSINSdt=−K4(XE4XE4Re⁡f)(SINSSINS+KS4)X     (23)
 MathType@MTEF@5@5@+=feaafiart1ev1aaatCvAUfKttLearuWrP9MDH5MBPbIqV92AaeXatLxBI9gBaebbnrfifHhDYfgasaacH8akY=wiFfYdH8Gipec8Eeeu0xXdbba9frFj0=OqFfea0dXdd9vqai=hGuQ8kuc9pgc9s8qqaq=dirpe0xb9q8qiLsFr0=vr0=vr0dc8meaabaqaciaacaGaaeqabaqabeGadaaakeaadaWcaaqaaiabdsgaKjabdofatnaaBaaaleaacqWGjbqscqWGobGtcqWGtbWuaeqaaaGcbaGaemizaqMaemiDaqhaaiabg2da9iabgkHiTiabdUealnaaBaaaleaacqaI0aanaeqaaOWaaeWaaeaadaWcaaqaaiabdIfaynaaBaaaleaacqWGfbqrcqaI0aanaeqaaaGcbaGaemiwaG1aa0baaSqaaiabdweafjabisda0aqaaiGbckfasjabcwgaLjabdAgaMbaaaaaakiaawIcacaGLPaaadaqadaqaamaalaaabaGaem4uam1aaSbaaSqaaiabdMeajjabd6eaojabdofatbqabaaakeaacqWGtbWudaWgaaWcbaGaemysaKKaemOta4Kaem4uamfabeaakiabgUcaRiabdUealnaaBaaaleaacqWGtbWucqaI0aanaeqaaaaaaOGaayjkaiaawMcaaiabdIfayjaaxMaacaWLjaWaaeWaaeaacqaIYaGmcqaIZaWmaiaawIcacaGLPaaaaaa@5BC9@

dXE1iXE1iRefdt=A1,ir1,i*−(μ+Beta1,i)XE1,iXE1,iRef     (24)
 MathType@MTEF@5@5@+=feaafiart1ev1aaatCvAUfKttLearuWrP9MDH5MBPbIqV92AaeXatLxBI9gBaebbnrfifHhDYfgasaacH8akY=wiFfYdH8Gipec8Eeeu0xXdbba9frFj0=OqFfea0dXdd9vqai=hGuQ8kuc9pgc9s8qqaq=dirpe0xb9q8qiLsFr0=vr0=vr0dc8meaabaqaciaacaGaaeqabaqabeGadaaakeaabaWaaSaaaeaacqqGKbazdaWcaaqaaiabbIfaynaaBaaaleaacqqGfbqrcqqGXaqmdaWgaaadbaGaeeyAaKgabeaaaSqabaaakeaacqqGybawdaqhaaWcbaGaemyrauKaeGymaeZaaSbaaWqaaiabbMgaPbqabaaaleaacqqGsbGucqqGLbqzcqqGMbGzaaaaaaGcbaGaeeizaqMaeeiDaqhaaiabg2da9iabdgeabnaaBaaaleaacqaIXaqmcqGGSaalcqWGPbqAaeqaaOGaemOCai3aa0baaSqaaiabigdaXiabcYcaSiabdMgaPbqaaiabcQcaQaaaaOGaeyOeI0YaaeWaaeaacqaH8oqBcqGHRaWkcqWGcbGqcqWGLbqzcqWG0baDcqWGHbqydaWgaaWcbaGaeGymaeJaeiilaWIaemyAaKgabeaaaOGaayjkaiaawMcaamaalaaabaGaemiwaG1aaSbaaSqaaiabbweafjabbgdaXiabcYcaSiabbMgaPbqabaaakeaacqWGybawdaqhaaWcbaGaeeyrauKaeeymaeJaeiilaWIaeeyAaKgabaGaeeOuaiLaeeyzauMaeeOzaygaaaaakiaaxMaacaWLjaWaaeWaaeaacqaIYaGmcqaI0aanaiaawIcacaGLPaaaaaa@6B24@

dXE2iXE2iRefdt=A2,ir2,i*−(μ+Beta2,i)XE2,iXE2,iRef     (25)
 MathType@MTEF@5@5@+=feaafiart1ev1aaatCvAUfKttLearuWrP9MDH5MBPbIqV92AaeXatLxBI9gBaebbnrfifHhDYfgasaacH8akY=wiFfYdH8Gipec8Eeeu0xXdbba9frFj0=OqFfea0dXdd9vqai=hGuQ8kuc9pgc9s8qqaq=dirpe0xb9q8qiLsFr0=vr0=vr0dc8meaabaqaciaacaGaaeqabaqabeGadaaakeaabaWaaSaaaeaacqqGKbazdaWcaaqaaiabbIfaynaaBaaaleaacqqGfbqrcqqGYaGmdaWgaaadbaGaeeyAaKgabeaaaSqabaaakeaacqqGybawdaqhaaWcbaGaemyrauKaeGOmaiZaaSbaaWqaaiabbMgaPbqabaaaleaacqqGsbGucqqGLbqzcqqGMbGzaaaaaaGcbaGaeeizaqMaeeiDaqhaaiabg2da9iabdgeabnaaBaaaleaacqaIYaGmcqGGSaalcqWGPbqAaeqaaOGaemOCai3aa0baaSqaaiabikdaYiabcYcaSiabdMgaPbqaaiabcQcaQaaaaOGaeyOeI0YaaeWaaeaacqaH8oqBcqGHRaWkcqWGcbGqcqWGLbqzcqWG0baDcqWGHbqydaWgaaWcbaGaeGOmaiJaeiilaWIaemyAaKgabeaaaOGaayjkaiaawMcaamaalaaabaGaemiwaG1aaSbaaSqaaiabbweafjabikdaYiabcYcaSiabbMgaPbqabaaakeaacqWGybawdaqhaaWcbaGaeeyrauKaeGOmaiJaeiilaWIaeeyAaKgabaGaeeOuaiLaeeyzauMaeeOzaygaaaaakiaaxMaacaWLjaWaaeWaaeaacqaIYaGmcqaI1aqnaiaawIcacaGLPaaaaaa@6B42@

dXE3XE3Refdt=A3r3*−(μ+Beta3)XE3XE3Ref     (26)
 MathType@MTEF@5@5@+=feaafiart1ev1aaatCvAUfKttLearuWrP9MDH5MBPbIqV92AaeXatLxBI9gBaebbnrfifHhDYfgasaacH8akY=wiFfYdH8Gipec8Eeeu0xXdbba9frFj0=OqFfea0dXdd9vqai=hGuQ8kuc9pgc9s8qqaq=dirpe0xb9q8qiLsFr0=vr0=vr0dc8meaabaqaciaacaGaaeqabaqabeGadaaakeaabaWaaSaaaeaacqqGKbazdaWcaaqaaiabbIfaynaaBaaaleaacqqGfbqrcqqGZaWmaeqaaaGcbaGaeeiwaG1aa0baaSqaaiabbweafjabbodaZaqaaiabbkfasjabbwgaLjabbAgaMbaaaaaakeaacqqGKbazcqqG0baDaaGaeyypa0Jaemyqae0aaSbaaSqaaiabiodaZaqabaGccqWGYbGCdaqhaaWcbaGaeG4mamdabaGaeiOkaOcaaaGccqGHsisldaqadaqaaiabeY7aTjabgUcaRiabdkeacjabdwgaLjabdsha0jabdggaHnaaBaaaleaacqaIZaWmaeqaaaGccaGLOaGaayzkaaWaaSaaaeaacqqGybawdaWgaaWcbaGaeeyrauKaee4mamdabeaaaOqaaiabbIfaynaaDaaaleaacqqGfbqrcqaIZaWmaeaacqqGsbGucqqGLbqzcqqGMbGzaaaaaOGaaCzcaiaaxMaadaqadaqaaiabikdaYiabiAda2aGaayjkaiaawMcaaaaa@5CF9@

dXE4XE4Refdt=(μ+Beta41+(NTw)d)−(μ+Beta4)XE4XE4Ref     (27)
 MathType@MTEF@5@5@+=feaafiart1ev1aaatCvAUfKttLearuWrP9MDH5MBPbIqV92AaeXatLxBI9gBaebbnrfifHhDYfgasaacH8akY=wiFfYdH8Gipec8Eeeu0xXdbba9frFj0=OqFfea0dXdd9vqai=hGuQ8kuc9pgc9s8qqaq=dirpe0xb9q8qiLsFr0=vr0=vr0dc8meaabaqaciaacaGaaeqabaqabeGadaaakeaabaWaaSaaaeaacqqGKbazdaWcaaqaaiabbIfaynaaBaaaleaacqqGfbqrcqqG0aanaeqaaaGcbaGaeeiwaG1aa0baaSqaaiabbweafjabisda0aqaaiabbkfasjabbwgaLjabbAgaMbaaaaaakeaacqqGKbazcqqG0baDaaGaeeypa0ZaaeWaaeaadaWcaaqaaiabeY7aTjabgUcaRiabdkeacjabdwgaLjabdsha0jabdggaHnaaBaaaleaacqaI0aanaeqaaaGcbaGaeGymaeJaey4kaSYaaeWaaeaadaWcaaqaaiabd6eaonaaBaaaleaacqWGubavaeqaaaGcbaGaem4DaChaaaGaayjkaiaawMcaamaaCaaaleqabaGaemizaqgaaaaaaOGaayjkaiaawMcaaaGaeyOeI0YaaeWaaeaacqaH8oqBcqGHRaWkcqWGcbGqcqWGLbqzcqWG0baDcqWGHbqydaWgaaWcbaGaeGinaqdabeaaaOGaayjkaiaawMcaamaalaaabaGaeeiwaG1aaSbaaSqaaiabbweafjabbsda0aqabaaakeaacqqGybawdaqhaaWcbaGaeeyrauKaeeinaqdabaGaeeOuaiLaeeyzauMaeeOzaygaaaaakiaaxMaacaWLjaWaaeWaaeaacqaIYaGmcqaI3aWnaiaawIcacaGLPaaaaaa@6AC6@

### Estimation of kinetic parameters

The kinetic parameter values were estimated by fitting the simulation profiles obtained by solving equations 16–22 to the corresponding dynamic profile determined experimentally. The estimated parameter values were optimized via a dynamic optimization algorithm "fmincon" available in the software MATLAB (Mathworks, Natick, MA). The routine is utilized to minimize the deviation between the experimental and the model-predicted values of the variables such as biomass, rifamycin B, *S*_*glc *_and *S*_*AAi*_. An analysis of variance (ANOVA) approach to regression analysis was applied for the estimation of deviation The method partitions the total sum of squares (SSTO) into the error sum of squares (SSE) and the regression sum of squares (SSR)[40].

## Competing interests

The author(s) declare that they have no competing interests.

## Authors' contributions

PMB performed most of the experiments and contributed to experiment design and manuscript preparation; SVS performed the amino acid analysis; DD contributed to mathematical modeling and simulations; PPW proposed the model, supervised the work and coordinated the manuscript preparation All authors read and approved the final manuscript.

## Nomenclature

SAAi
 MathType@MTEF@5@5@+=feaafiart1ev1aaatCvAUfKttLearuWrP9MDH5MBPbIqV92AaeXatLxBI9gBaebbnrfifHhDYfgasaacH8akY=wiFfYdH8Gipec8Eeeu0xXdbba9frFj0=OqFfea0dXdd9vqai=hGuQ8kuc9pgc9s8qqaq=dirpe0xb9q8qiLsFr0=vr0=vr0dc8meaabaqaciaacaGaaeqabaqabeGadaaakeaacqWGtbWudaWgaaWcbaGaemyqaeKaemyqae0aaSbaaWqaaiabdMgaPbqabaaaleqaaaaa@31B0@ amino acids (mole.L^-1^)

*S*_*glc *_Glucose (mmole.L^-1^)

*S*_*amm *_Ammonia, (mmole.L^-1^)

XE1i
 MathType@MTEF@5@5@+=feaafiart1ev1aaatCvAUfKttLearuWrP9MDH5MBPbIqV92AaeXatLxBI9gBaebbnrfifHhDYfgasaacH8akY=wiFfYdH8Gipec8Eeeu0xXdbba9frFj0=OqFfea0dXdd9vqai=hGuQ8kuc9pgc9s8qqaq=dirpe0xb9q8qiLsFr0=vr0=vr0dc8meaabaqaciaacaGaaeqabaqabeGadaaakeaacqWGybawdaWgaaWcbaGaemyrauKaeGymaeZaaSbaaWqaaiabdMgaPbqabaaaleqaaaaa@31A7@ Concentration of the enzyme responsible for uptake of amino acid as sole source of carbon and nitrogen, moles.L^-1^

XE2i
 MathType@MTEF@5@5@+=feaafiart1ev1aaatCvAUfKttLearuWrP9MDH5MBPbIqV92AaeXatLxBI9gBaebbnrfifHhDYfgasaacH8akY=wiFfYdH8Gipec8Eeeu0xXdbba9frFj0=OqFfea0dXdd9vqai=hGuQ8kuc9pgc9s8qqaq=dirpe0xb9q8qiLsFr0=vr0=vr0dc8meaabaqaciaacaGaaeqabaqabeGadaaakeaacqWGybawdaWgaaWcbaGaemyrauKaeGOmaiZaaSbaaWqaaiabdMgaPbqabaaaleqaaaaa@31A9@ Concentration of the enzyme responsible for uptake of amino acid as nitrogen source and glucose as carbon source., moles.L^-1^

XE3i
 MathType@MTEF@5@5@+=feaafiart1ev1aaatCvAUfKttLearuWrP9MDH5MBPbIqV92AaeXatLxBI9gBaebbnrfifHhDYfgasaacH8akY=wiFfYdH8Gipec8Eeeu0xXdbba9frFj0=OqFfea0dXdd9vqai=hGuQ8kuc9pgc9s8qqaq=dirpe0xb9q8qiLsFr0=vr0=vr0dc8meaabaqaciaacaGaaeqabaqabeGadaaakeaacqWGybawdaWgaaWcbaGaemyrauKaeG4mamZaaSbaaWqaaiabdMgaPbqabaaaleqaaaaa@31AB@ Concentration of the enzyme responsible for uptake of ammonia as nitrogen source and glucose as carbon source, moles.L^-1^

*S*_*INS *_Insoluble nitrogen source, moles.L^-1^

*X*_*E*4 _Concentration of hydrolytic enzyme, moles.L^-1^

KS1i
 MathType@MTEF@5@5@+=feaafiart1ev1aaatCvAUfKttLearuWrP9MDH5MBPbIqV92AaeXatLxBI9gBaebbnrfifHhDYfgasaacH8akY=wiFfYdH8Gipec8Eeeu0xXdbba9frFj0=OqFfea0dXdd9vqai=hGuQ8kuc9pgc9s8qqaq=dirpe0xb9q8qiLsFr0=vr0=vr0dc8meaabaqaciaacaGaaeqabaqabeGadaaakeaacqWGlbWsdaWgaaWcbaGaem4uamLaeGymaeZaaSbaaWqaaiabdMgaPbqabaaaleqaaaaa@31A9@ Substrate half saturation constant for substrate SAAi
 MathType@MTEF@5@5@+=feaafiart1ev1aaatCvAUfKttLearuWrP9MDH5MBPbIqV92AaeXatLxBI9gBaebbnrfifHhDYfgasaacH8akY=wiFfYdH8Gipec8Eeeu0xXdbba9frFj0=OqFfea0dXdd9vqai=hGuQ8kuc9pgc9s8qqaq=dirpe0xb9q8qiLsFr0=vr0=vr0dc8meaabaqaciaacaGaaeqabaqabeGadaaakeaacqWGtbWudaWgaaWcbaGaemyqaeKaemyqae0aaSbaaWqaaiabdMgaPbqabaaaleqaaaaa@31B0@ (substrate combination: amino acid only), moles.L^-1^

KI1i
 MathType@MTEF@5@5@+=feaafiart1ev1aaatCvAUfKttLearuWrP9MDH5MBPbIqV92AaeXatLxBI9gBaebbnrfifHhDYfgasaacH8akY=wiFfYdH8Gipec8Eeeu0xXdbba9frFj0=OqFfea0dXdd9vqai=hGuQ8kuc9pgc9s8qqaq=dirpe0xb9q8qiLsFr0=vr0=vr0dc8meaabaqaciaacaGaaeqabaqabeGadaaakeaacqWGlbWsdaWgaaWcbaGaemysaKKaeGymaeZaaSbaaWqaaiabdMgaPbqabaaaleqaaaaa@3195@ Substrate inhibition constant for substrate SAAi
 MathType@MTEF@5@5@+=feaafiart1ev1aaatCvAUfKttLearuWrP9MDH5MBPbIqV92AaeXatLxBI9gBaebbnrfifHhDYfgasaacH8akY=wiFfYdH8Gipec8Eeeu0xXdbba9frFj0=OqFfea0dXdd9vqai=hGuQ8kuc9pgc9s8qqaq=dirpe0xb9q8qiLsFr0=vr0=vr0dc8meaabaqaciaacaGaaeqabaqabeGadaaakeaacqWGtbWudaWgaaWcbaGaemyqaeKaemyqae0aaSbaaWqaaiabdMgaPbqabaaaleqaaaaa@31B0@ (substrate combination: amino acid only), moles.L^-1^

*k*_*NT*1,*i *_Nitrogen catabolite repression for substrate i, (substrate combination: amino acid only), moles.L^-1^

Ks_2,*i *_Substrate half saturation constant for substrate SAAi
 MathType@MTEF@5@5@+=feaafiart1ev1aaatCvAUfKttLearuWrP9MDH5MBPbIqV92AaeXatLxBI9gBaebbnrfifHhDYfgasaacH8akY=wiFfYdH8Gipec8Eeeu0xXdbba9frFj0=OqFfea0dXdd9vqai=hGuQ8kuc9pgc9s8qqaq=dirpe0xb9q8qiLsFr0=vr0=vr0dc8meaabaqaciaacaGaaeqabaqabeGadaaakeaacqWGtbWudaWgaaWcbaGaemyqaeKaemyqae0aaSbaaWqaaiabdMgaPbqabaaaleqaaaaa@31B0@ (substrate combination: amino acid and glucose), moles.L^-1^

**Ks_2,2 _**Substrate half saturation constant for substrate *S*_*glc *_(substrate combination: amino acid and glucose), moles.L^-1^

**K_I 2, i _**Substrate inhibition constant for substrate SAAi
 MathType@MTEF@5@5@+=feaafiart1ev1aaatCvAUfKttLearuWrP9MDH5MBPbIqV92AaeXatLxBI9gBaebbnrfifHhDYfgasaacH8akY=wiFfYdH8Gipec8Eeeu0xXdbba9frFj0=OqFfea0dXdd9vqai=hGuQ8kuc9pgc9s8qqaq=dirpe0xb9q8qiLsFr0=vr0=vr0dc8meaabaqaciaacaGaaeqabaqabeGadaaakeaacqWGtbWudaWgaaWcbaGaemyqaeKaemyqae0aaSbaaWqaaiabdMgaPbqabaaaleqaaaaa@31B0@ (substrate combination: amino acid and glucose), moles.L^-1^

**K_I 2, 2 _**Substrate inhibition constant for substrate *S*_*glc *_(substrate combination: amino acid and glucose), moles.L^-1^

**Ks_3,3 _**Substrate half saturation constant for substrate *S*_*amm *_(substrate combination: ammonia and glucose), moles.L^-1^

**K_I 3,3 _**Substrate inhibitionconstant for substrate *S*_*amm *_(substrate combination: ammonia and glucose), moles.L^-1^

**Ks_3,2 _**Substrate half saturation constant for substrate *S*_*glc *_(substrate combination: ammonia and glucose), moles.L^-1^

**K_I 3,2 _**Substrate inhibition constant for substrate *S*_*glc *_(substrate combination: ammonia and glucose), moles.L^-1^

*μ*_1,i _Specific growth rate on S_*AA*,*i*_.(h^-1^)

*μ*_1,i_^max ^Maximum specific growth rate on SAAi
 MathType@MTEF@5@5@+=feaafiart1ev1aaatCvAUfKttLearuWrP9MDH5MBPbIqV92AaeXatLxBI9gBaebbnrfifHhDYfgasaacH8akY=wiFfYdH8Gipec8Eeeu0xXdbba9frFj0=OqFfea0dXdd9vqai=hGuQ8kuc9pgc9s8qqaq=dirpe0xb9q8qiLsFr0=vr0=vr0dc8meaabaqaciaacaGaaeqabaqabeGadaaakeaacqWGtbWudaWgaaWcbaGaemyqaeKaemyqae0aaSbaaWqaaiabdMgaPbqabaaaleqaaaaa@31B0@.(h^-1^)

*μ*_2,i _Specific growth rate on SAAi
 MathType@MTEF@5@5@+=feaafiart1ev1aaatCvAUfKttLearuWrP9MDH5MBPbIqV92AaeXatLxBI9gBaebbnrfifHhDYfgasaacH8akY=wiFfYdH8Gipec8Eeeu0xXdbba9frFj0=OqFfea0dXdd9vqai=hGuQ8kuc9pgc9s8qqaq=dirpe0xb9q8qiLsFr0=vr0=vr0dc8meaabaqaciaacaGaaeqabaqabeGadaaakeaacqWGtbWudaWgaaWcbaGaemyqaeKaemyqae0aaSbaaWqaaiabdMgaPbqabaaaleqaaaaa@31B0@ and *S*_*glc*_, (h^-1^)

*μ*_2,i_^max ^Maximum specific growth rate on SAAi
 MathType@MTEF@5@5@+=feaafiart1ev1aaatCvAUfKttLearuWrP9MDH5MBPbIqV92AaeXatLxBI9gBaebbnrfifHhDYfgasaacH8akY=wiFfYdH8Gipec8Eeeu0xXdbba9frFj0=OqFfea0dXdd9vqai=hGuQ8kuc9pgc9s8qqaq=dirpe0xb9q8qiLsFr0=vr0=vr0dc8meaabaqaciaacaGaaeqabaqabeGadaaakeaacqWGtbWudaWgaaWcbaGaemyqaeKaemyqae0aaSbaaWqaaiabdMgaPbqabaaaleqaaaaa@31B0@ and *S*_*glc*_, (h^-1^)

*μ*_3 _Specific growth rate on *S*_*amm*_, (h^-1^)

*μ*_3_^max ^Maximum specific growth rate on *S*_*amm*_, (h^-1^)

*q*_*p*2,*i *_Specific product formation rate on SAAi
 MathType@MTEF@5@5@+=feaafiart1ev1aaatCvAUfKttLearuWrP9MDH5MBPbIqV92AaeXatLxBI9gBaebbnrfifHhDYfgasaacH8akY=wiFfYdH8Gipec8Eeeu0xXdbba9frFj0=OqFfea0dXdd9vqai=hGuQ8kuc9pgc9s8qqaq=dirpe0xb9q8qiLsFr0=vr0=vr0dc8meaabaqaciaacaGaaeqabaqabeGadaaakeaacqWGtbWudaWgaaWcbaGaemyqaeKaemyqae0aaSbaaWqaaiabdMgaPbqabaaaleqaaaaa@31B0@ and *S*_*glc*_, (h^-1^)

*q*_*p*3 _Specific product formation rate on *S*_*glc *_and *S*_*amm*_, (h^-1^)

*q*_*p*3_^max ^Maximum specific product formation rate on *S*_*glc *_and *S*_*amm*_, (h^-1^)

Y_i,j _Stoichiometric coefficient of substrate j in reaction i. (moles of j.C-mole of biomass^-1^)
